# Liquid jet capabilities for ultrafast chemistry at the SwissFEL Alvra instrument

**DOI:** 10.1107/S1600577526003735

**Published:** 2026-05-08

**Authors:** Claudio Cirelli, Emma Victoria Beale, Florian Dworkowski, Victoria Kabanova, Sven Augustin, Gregor Knopp, Chris Milne, Christoph Bostedt, Philip J. M. Johnson, Camila Bacellar

**Affiliations:** a Paul Scherrer Institute CH-5232Villigen PSI Switzerland; b LUXS Laboratory for Ultrafast X-ray Sciences, Institute of Chemical Sciences and Engineering École Polytechnique Fédérale de Lausanne (EPFL) CH-1015Lausanne Switzerland; DESY, Germany

**Keywords:** X-ray free-electron lasers, ultrafast photochemistry, time-resolved X-ray spectroscopy, time-resolved X-ray solution scattering

## Abstract

The Alvra experimental station at the Swiss X-ray free-electron laser, SwissFEL, investigates ultrafast dynamics in chemical and biological systems using X-ray scattering and spectroscopy techniques. A key feature of Alvra is its unique capability to perform time-resolved X-ray emission and resonant inelastic X-ray scattering in the tender X-ray regime, currently not available at other XFEL endstations, combined with simultaneous access to X-ray absorption spectroscopy and X-ray solution scattering. Together with sub-35 fs time resolution enabled by advanced timing diagnostics, this positions Alvra as a versatile instrument for ultrafast chemical dynamics in the liquid phase. Here we cover the various technical aspects of the beamline and experimental station, and present some examples of experimental results measured during the first years of SwissFEL commissioning and user operation.

## Abstract 2

The technical aspects of the Alvra endstation are outlined and its scientific capabilities are demonstrated with commissioning results obtained during the early years of SwissFEL’s operation.

## 1. Introduction

Time-resolved X-ray spectroscopy and scattering are invaluable techniques for the study of chemical dynamics, enabling the direct visualization of structural and electronic changes during photoactivated chemical reactions and biological processes (Li *et al.*, 2017 ▸; Consani *et al.*, 2014 ▸; Scholes *et al.*, 2017 ▸; Chen *et al.*, 1997 ▸; Wernet *et al.*, 2015 ▸; Öström *et al.*, 2015 ▸; Mara *et al.*, 2017 ▸; Kjaer *et al.*, 2018 ▸). In the last 15 years, since the advent of X-ray free-electron lasers (XFELs), pulses of unprecedented peak brightness and ultrashort duration across a large range of photon energies have made it possible to observe ultrafast processes with previously inaccessible temporal resolution when employing X-rays. After the successful proof-of-principle experiments at third-generation synchrotron light sources (Huse *et al.*, 2011 ▸; Vankó *et al.*, 2006 ▸; Vankó *et al.*, 2013 ▸; Saes *et al.*, 2003 ▸), photon-hungry techniques such as *K*_α_, *K*_β_ and valence-to-core (VtC) X-ray emission spectroscopy (XES) and resonant inelastic X-ray scattering (RIXS) have also been extended into the time domain and are now routine measurements at XFELs.

These rapid developments have paved the way for the comprehensive understanding of ultrafast processes, such as spin crossover (SCO) dynamics (Bressler *et al.*, 2009 ▸; Auböck & Chergui, 2015 ▸), intramolecular charge and energy transfer, excited state structural deformations, among others, in both model systems and complex chemical species in solution (Bressler *et al.*, 2009 ▸; Lemke *et al.*, 2013 ▸; Huse *et al.*, 2011 ▸; Yong *et al.*, 2021 ▸; Gaffney, 2021 ▸). For instance, Zhang *et al.* (Zhang *et al.*, 2014 ▸) used time-resolved *K*_β_ XES to observe SCO dynamics in a model iron complex, providing direct evidence of the interplay between electronic and structural changes during the photoinduced transition from a low spin (LS) state to a high spin (HS) state, thus establishing a benchmark for the identification of high-spin intermediates.

Combining techniques, such as X-ray absorption spectroscopy (XAS) and XES, has also proven to be a powerful approach to elucidate the details of dynamics in chemical and biologically relevant systems (Dell’Angela *et al.*, 2013 ▸; Kunnus *et al.*, 2016 ▸). At the Alvra endstation, this was employed in the study of cytochrome C, where ultrafast XAS and XES experiments have demonstrated that photoexcitation of the ferric form of the protein induces a cascade through excited spin states of iron, leading to doming of the heme plane (Bacellar *et al.*, 2020 ▸; Bacellar *et al.*, 2023 ▸), challenging previous assumptions about electronic relaxation in ferric hemes (Mara *et al.*, 2017 ▸). Photo-triggered chemical reactions in the electronic ground state have also been closely followed with X-ray spectroscopy techniques. Recent studies also performed at Alvra have used ultrafast XAS and RIXS to track charge-transfer interactions during alkane C–H activation by a rhodium complex, revealing how alkane-to-metal donation and metal-to-alkane back-donation influence complex stability and bond cleavage (Jay *et al.*, 2023 ▸; Banerjee *et al.*, 2024 ▸; Lim *et al.*, 2025 ▸).

The limits of what is achievable in terms of signal-to-noise ratio (SNR) and time resolution are constantly evolving. To fully benefit from the unique beam properties of XFELs, specialized beamlines have developed around specific scientific and technical needs. Within this landscape, SwissFEL came into user operation in 2019 (Prat *et al.*, 2020 ▸; Milne *et al.*, 2017*a* ▸). SwissFEL currently operates two branches: the hard X-ray branch, Aramis, with three experimental stations (Alvra, Bernina and Cristallina), and the soft X-ray branch, Athos, with two (Furka and Maloja). This paper describes the cylindrical liquid jet capabilities and future developments at the Alvra experimental station, which is focused on ultrafast chemistry and biology experiments using X-ray techniques. We first cover a brief overview of the facility, beamline components, and photon diagnostics elements. We also provide details on the experimental station sample environments, detector, and the various auxiliary pieces of hardware available for user experiments at Alvra. Finally, we give representative examples of measurements performed at the experimental station, thereby demonstrating the different experimental configurations available.

## 2. The SwissFEL facility

The SwissFEL XFEL facility is located at the Paul Scherrer Institute (PSI), the Swiss national laboratory center for large-scale user facilities. It is the latest accelerator-based facility to come into operation at PSI and is one of the most recent XFELs to come into user operation. The linear accelerator of SwissFEL generated its first XFEL radiation in November 2017. After one year of commissioning and pilot phase experiments, Aramis started user operation in January 2019. To date, it has completed over 100 user experiments distributed across its three experimental stations. In 2021, the Athos branch was opened to the user community and began operation with two experimental stations, with a third under commissioning at the time of publication.

### 2.1. X-ray source

The key features of SwissFEL are its stability, compactness and consequent reduced building and operation costs (Prat *et al.*, 2020 ▸). The three-stage copper linear accelerator of SwissFEL consists of an S-band photo-injector and three C-band booster sections that increase the electron energy up to a maximum of 5.8 GeV at a nominal electron bunch charge of 200 pC. Peak currents of almost 3 kA are reached by compressing the bunch charge with a two-stage magnetic bunch compressor (BC) located after the injector (BC1) and after the first linac (BC2).

The design of the machine allows for the acceleration of two electron bunches within the radiofrequency (RF) pulse duration of 23 ns. These two bunches can be sent to the two branches of the accelerator using a fast electron kicker, simultaneously driving two XFELs operated in self-amplified spontaneous emission (SASE) mode at 100 Hz: the hard X-ray Aramis undulator (0.1–0.7 nm, corresponding to about 1.8–13 keV) at 5.8 GeV and the soft X-ray Athos undulator (0.65–5 nm, *i.e.* 0.26–1.9 keV) at 3.0 GeV.

The Aramis branch utilizes variable-gap in-vacuum undulators, which feature the shortest period (15 mm) among all XFEL facilities, to generate linearly, horizontally polarized X-rays with a bandwidth ≤ 0.2% for any photon energy over the entire 2–12.4 keV energy range in normal SASE mode. A special mode of operation, delivering large bandwidth pulses with a spectral width up to 3.5% bandwidth, has also recently been demonstrated (Reiche *et al.*, 2023 ▸; Nass *et al.*, 2021 ▸; Fadini *et al.*, 2020 ▸; Juranić *et al.*, 2024 ▸).

Electron beam arrival moninors (BAMs) are installed along the electron accelerator. The most relevant ones are located just after each BC and at the end of the undulator line, with the purpose of providing shot-to-shot electron bunch arrival time information with an accuracy of the order of a few femtoseconds (Löhl *et al.*, 2010 ▸). In addition, a post-undulator passive wakefield streaker is used to measure the single-shot temporal profile of both the electron and the XFEL pulses with femtosecond resolution (Bettoni *et al.*, 2016 ▸; Dijkstal *et al.*, 2022 ▸). This approach consists of streaking the electron beam using the wakefields of a corrugated structure and represents an alternative approach to the more established method of using an X-band (∼12 GHz) RF transverse deflector (TD) for streaking (Ding *et al.*, 2011 ▸; Behrens *et al.*, 2014 ▸). Compared with the TD method, passive streaking offers inherent stability against arrival-time jitter and requires simpler, more cost-effective hardware.

### 2.2. X-ray optics and diagnostics

**Figure 1:**
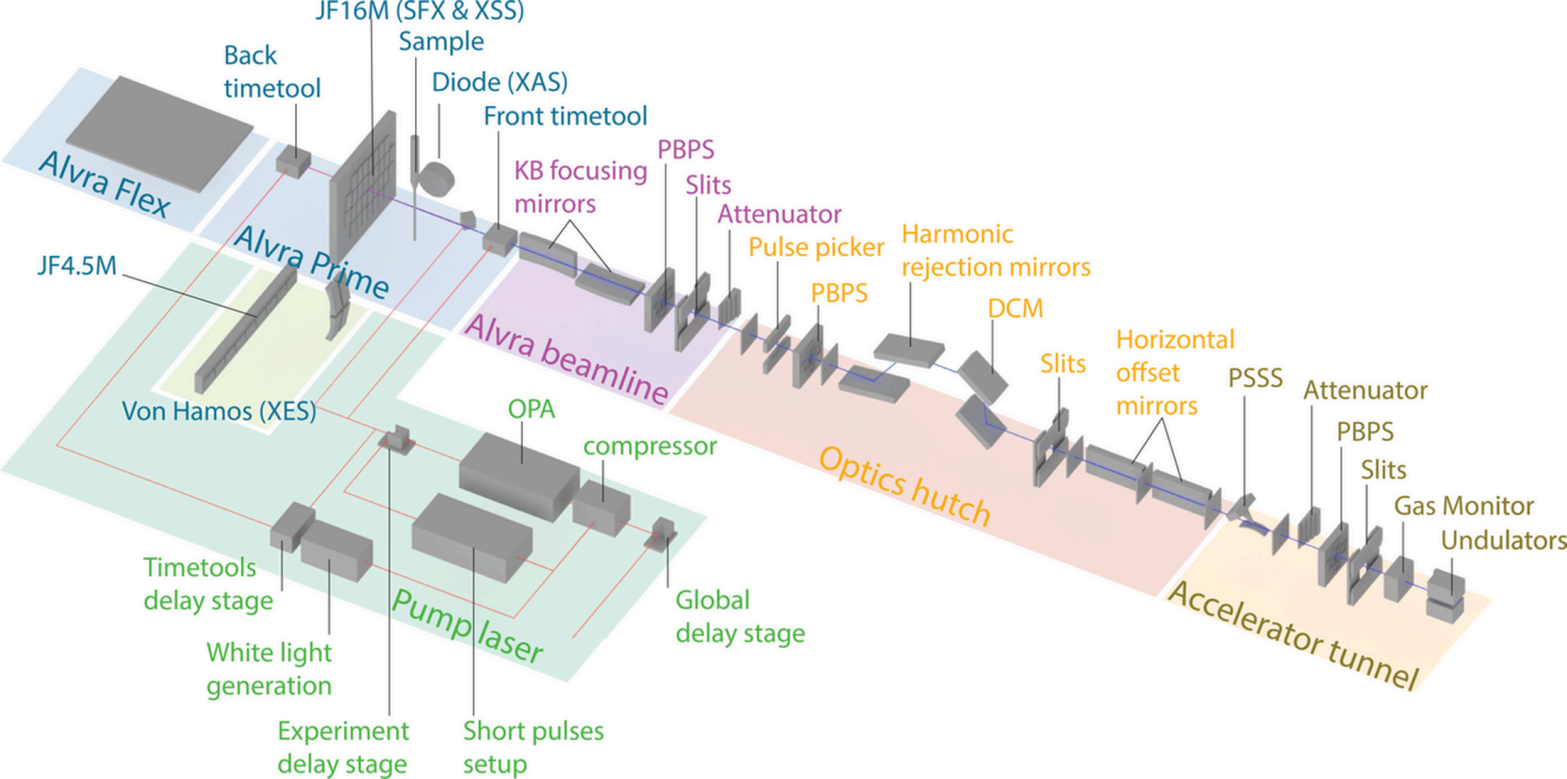
Schematic layout of the Aramis Alvra beamline. The X-ray optics consists of a set of offset mirrors, a double-crystal monochromator (DCM) and a pair of focusing (KB) mirrors. The XFEL beam trajectory is defined by multiple aperture sets positioned along the beamline. The beam profile and size can be monitored at several locations using PPRMs based on Ce:YAG scintillators. The incident X-ray flux is controlled using solid-state diamond and silicon attenuators. Single-shot XFEL pulse position and intensity are measured non-destructively in the accelerator tunnel using a gas-based ionization monitor and at various points along the beamline using photon beam position sensors (PBPSs) relying on X-ray back-scattering from thin solid targets. Reduced repetition rates or user-defined XFEL pulse sequences can be selected using a pulse picker.

Fig. 1 ▸ shows a schematic layout of the Alvra instrument and beamline, part of the Aramis hard X-ray branch. It illustrates the main X-ray optics and diagnostic elements employed to steer and characterize the XFEL beam from the undulators to the experimental station (Follath *et al.*, 2016 ▸).

Immediately following the Aramis undulator section and the electron beam dump, a high-transmission ionization chamber (gas monitor) passively measures the intensity and pointing of the SwissFEL pulses in a single-shot manner. If properly calibrated, this gas monitor can operate over the entire energy range of the XFEL and can quantify the absolute X-ray intensity for each pulse with an accuracy of about 10% (Juranić *et al.*, 2018 ▸).

The last device installed in the accelerator tunnel is a single shot X-ray spectrometer (PSSS). This device measures the single shot spectra of the XFEL for photon energies over the range of 4–13 keV, with a resolution Δ*E*/*E* = (2–5) × 10^−5^ over a bandwidth of 0.5% (Rehanek *et al.*, 2017 ▸). It utilizes a thin diamond transmission grating to diffract light into multiple orders in the horizontal plane (David *et al.*, 2021 ▸). The first-order diffracted beam is dispersed by a bent Si crystal onto a Ce:YAG scintillator and imaged by a fast frame imaging camera.

The XFEL beam mode and size can be measured along the beamline with a series of Photon Profile Monitors (PPRMs), based on Ce:YAG scintillators imaged by a camera. These PPRMs are are largely destructive and are therefore used predominantly during the machine setup and alignment to check the beam mode and trajectory. Three sets of slits located at various distances from the end of the undulator line (44, 104 and 120 m, respectively) define the default X-ray trajectory. The XFEL pulse position and intensity can be measured non-destructively with intensity and position monitors [photon beam position sensors (PBPSs) (Tono *et al.*, 2011 ▸)] placed at various locations along the beamline. These devices provide an online diagnostic and can be used as feedback sensors to correct long-term pointing and intensity drifts.

Solid state attenuators (based on silicon and diamond filters) can be used to reduce the XFEL intensity. A first attenuator unit is located just after the gas monitor on the part of the beamline shared between the three Aramis instruments. A second unit is placed in each of the experimental hutches a few meters before the sample interaction point.

The Aramis optical beamline design enables efficient switching of the XFEL beam between the three experimental stations Alvra, Bernina and Cristallina. The Alvra beamline propagates on the left in the horizontal direction from the straight trajectory defined by the Aramis undulators. Two consecutive offset mirrors deflect the beam horizontally for a total deflection of about 6 mrad (3 mrad from each mirror). In order to increase the reflectivity over the full Aramis photon energy range and beyond, the mirrors are coated with low-*Z* (10 nm B_4_C on top of 36 nm SiC) and mid-*Z* (15 nm B_4_C on top of 20 nm Mo) bi-layers. At a deflection angle of 3 mrad, these coatings extend the X-ray reflectivity cutoff relative to bare Si from approximately 10–13 keV to almost 20 keV.

**Figure 2:**
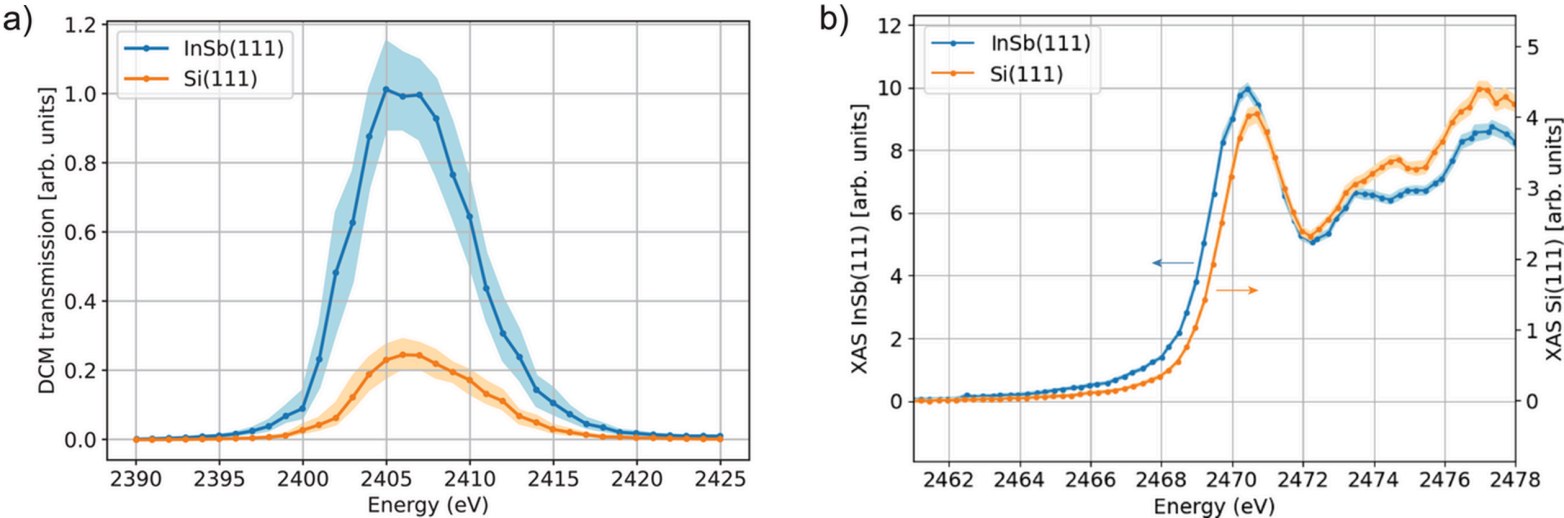
(*a*) Averaged SASE spectrum measured after the DCM aligned with Si(111) (orange solid line) and InSb(111) (blue solid line) crystal pairs, showing the fourfold increased transmission. (*b*) XANES ground state spectra of the reference sample sodium thiosulfate (Na_2_S_2_O_3_) measured at the sulfur *K*-edge: the use of the lower resolution InSb(111) crystal pair broadens by about 20% some of the spectroscopic features.

A double-crystal monochromator (DCM) can be inserted into the X-ray beam path to filter monochromatic light at a chosen wavelength, out of the full SASE XFEL spectrum. The DCM has three pairs of crystals mounted on a common Bragg rotation stage covering a range of incidence angles between 5° and 80°. With the standard set of Si(111) crystals, a resolving power *E*/Δ*E* ≃ 10000 (relative bandwidth ≤ 0.01%) is achieved over the entire Aramis photon energy range. An additional pair of high energy resolution crystals Si(311) can be used to increase the resolving power to *E*/Δ*E* ≃ 40000 (relative bandwidth 0.003%). Alternatively, a set of InSb(111) crystal pair can be used for high photon throughput at the cost of lower resolving power *E*/Δ*E* ≃ 3000 (relative bandwidth 0.04%). Fig. 2 ▸ compares the transmission through the DCM when pairs of Si(111) or InSb(111) crystals are used. Fig. 2 ▸(*a*) demonstrates the expected four-fold increase in flux. The lower energy resolution for the higher transmission crystal pair can be seen in Fig. 2 ▸(*b*) where X-ray absorption near-edge structure (XANES) spectra measured using each pair of crystals for the reference sample sodium thiosulfate Na_2_S_2_O_3_ at the S *K*-edge are compared. The width of the white-line peak at 2470.5 eV is approximately 20% broader when the InSb(111) pair of crystals are used.

The DCM geometry with translation perpendicular to the second crystal surface enables an adjustable beam offset up to 32 mm. A set of additional vertical offset mirrors is used to align the trajectory of the monochromatic beam with that of the full SASE pink beam. In the tender X-ray regime, these mirrors – which have part of their surface left as uncoated silicon – can also be used as harmonic rejection mirrors (HRM).

An X-ray pulse picker is installed in the beamline downstream of the HRM. This device is designed to selectively transmit specific XFEL pulses at a frequency lower than the standard 100 Hz SwissFEL repetition rate, or according to a user-defined pulse sequence. In addition to this continuous, ‘low-frequency’ mode, the pulse picker can also operate in a ‘burst’ mode, in which one or more XFEL pulses are transmitted on demand.

The last X-ray optical element before the sample interaction chamber is a pair of bendable Kirkpatrick–Baez (KB) focusing mirrors (Kirkpatrick & Baez, 1948 ▸) located at 1.4 m (horizontal axis) and 2.2 m (vertical axis) upstream of the nominal sample interaction point.

The KB mirrors enable achromatic focusing of the XFEL beam with high transmission across the entire Aramis energy range, thanks to their operation at grazing incidence angles. These angles can be set to achieve a total deflection between 8 and 12 mrad. At the primary sample interaction point, the XFEL spot size can be independently adjusted in the horizontal and vertical directions, with achieved beam sizes ranging from 1 µm (tightest focus achieved at 12 keV photon energy) up to ∼800 µm (collimated beam).

## 3. The Alvra instrument

### 3.1. Alvra Prime and Alvra Flex

**Figure 3:**
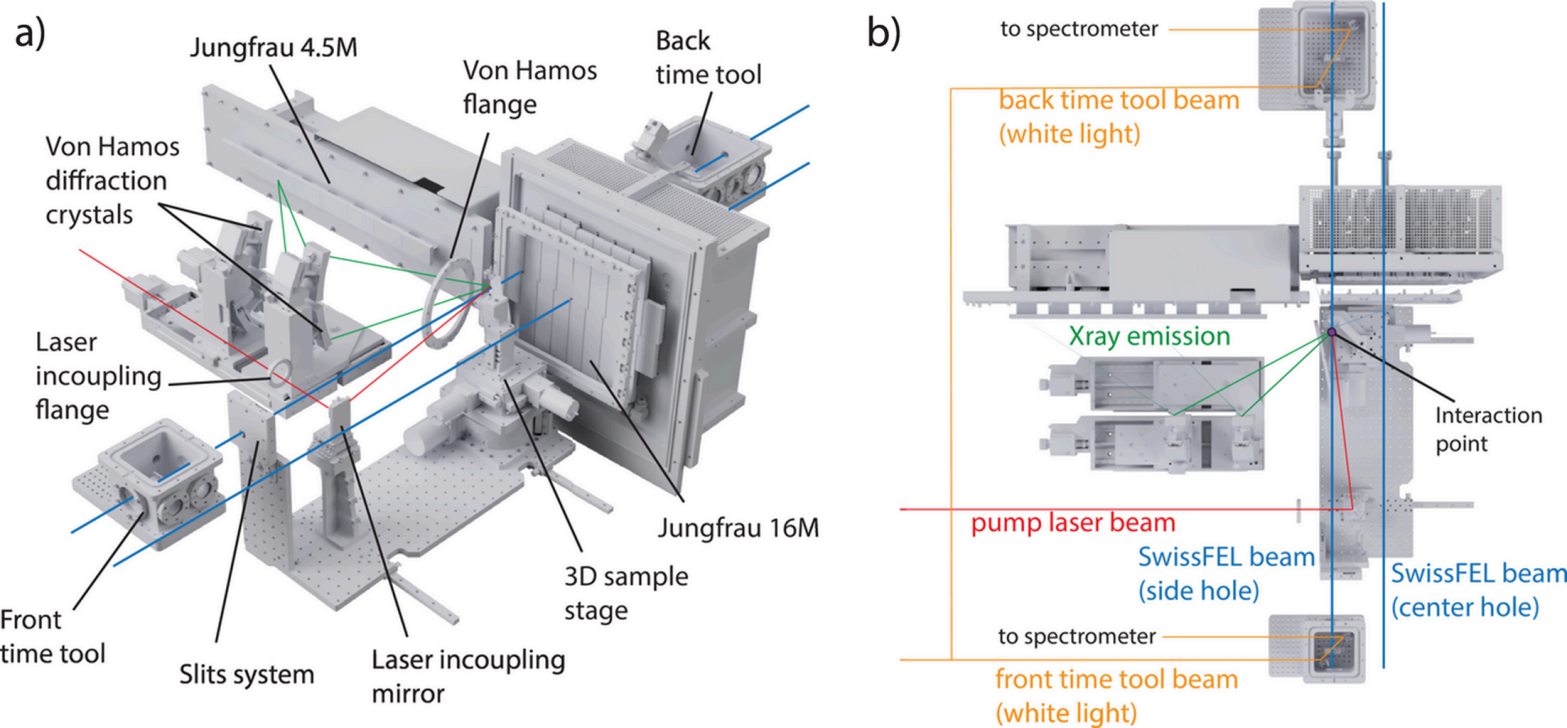
Isometric (*a*) and top (*b*) view of the Alvra Prime endstation, equipped with two JUNGFRAU 2D detectors for scattering and emission experiments.

The Alvra instrument consists of two endstations designed for time-resolved X-ray scattering and spectroscopy experiments: Alvra Prime and Alvra Flex. Alvra Prime operates under vacuum, helium or nitrogen atmosphere and is equipped with a large 2D 16M JUNGFRAU (Mozzanica *et al.*, 2018 ▸) scattering detector. Additionally, it incorporates a short-radius (25 cm) four-crystal X-ray emission spectrometer assembled in a cylindrical von Hamos geometry (Szlachetko *et al.*, 2012 ▸), which images emission photons onto a 4.5M JUNGFRAU detector (see Fig. 3 ▸). The spectrometer is equipped with a set of interchangeable diffraction crystals that can be installed on both Prime and Flex and are selected according to the target photon energy, covering the full Aramis energy range (Milne *et al.*, 2017*b* ▸).

The Prime chamber is mounted on a motorized rail that enables movement perpendicular to the XFEL beam. This allows the 16M detector to be positioned in two distinct scattering geometries [Fig. 3 ▸(*b*)]: one in which the XFEL beam is aligned with the center of the detector and a second in which the beam is shifted towards the left edge of the JUNGFRAU 16M detector by 12.5 cm. The former configuration allows fully symmetric scattering measurements, with an upper limit of momentum transfer, *q*, of approximately 7 Å^−1^ at 13 keV. The latter configuration enables access to higher values (*q* ≃ 8 Å^−1^ at 13 keV), albeit with a radial profile asymmetric in the horizontal direction. Moreover, such an arrangement is compatible with a wide range of Bragg angles accessible in the von Hamos emission spectrometer and enables simultaneous experiments combining both X-ray scattering and emission measurements.

Two additional small chambers are installed along the beamline before and after the Prime chamber, see Fig. 3 ▸. They contain a series of solid targets used for a spectral encoder time tool (Bionta *et al.*, 2011 ▸; Bionta *et al.*, 2014 ▸; Harmand *et al.*, 2013 ▸). These devices enable single-shot, non-destructive measurements of the difference in arrival time between the experimental laser and the X-ray pulses, described in detail in Section 3.2.3.

At the interaction region, different types of sample injectors can be installed, including some specifically designed for serial femtosecond crystallography (SFX) sample delivery, such as a high viscosity extruder (HVE) (Weierstall *et al.*, 2014 ▸). A recirculating liquid jet system is used for the delivery of solutions and other liquid samples, including nanoparticle suspensions at both the Prime and Flex endstations. Cylindrical jets of varying thicknesses (15, 25, 50, 75, 100 and 200 µm) as well as thin (≤30 µm) and thick (≥100 µm) flat-sheet jets are available and a drop-on-demand system is under commissioning at the time of publication. When using the cylindrical or flat-sheet jets, samples are pumped through the nozzle using a combination of high-performance liquid chromatography (HPLC) or syringe pumps (depending on the application) at average flow rates of 0.5–3.0 ml min^−1^. Samples are then collected in a Teflon catcher tube and pumped back into their reservoir using peristaltic pumps. This system can be completely closed to the atmosphere, enabling the handling of oxygen- and water-sensitive samples. Most experiments make use of the cylindrical liquid jets and can be run with sample aliquots of 20–30 ml. However, for specific applications these jets can be run with volumes as low as 5 ml. A mobile UV-Vis spectrometer loop is also available to monitor the sample constitution in real time during the experiments.

The Alvra Flex endstation is located downstream of Prime. It features a motorized table and supports the installation of user-supplied experimental chambers. Flex is equipped with a versatile X-ray emission spectrometer that can be configured in both vertical and horizontal geometries. The spectrometer employs up to three bent diffraction crystals to achieve a large solid-angle acceptance and can cover Bragg angles in a range from approximately 40° to 85°. The emitted radiation can be recorded on a 0.5M JUNGFRAU ‘stripsel’ detector, which offers a reduced pixel size of 25 µm × 225 µm along the dispersion direction, enabling enhanced energy resolution.

### 3.2. Experimental laser capabilities and timing diagnostics

To initiate the photochemistry or photoinduced biological processes under study at the Alvra Prime or Flex endstation, an experimental laser system providing femtosecond pump pulses with wavelengths tunable from the UV to the near IR (240 nm to 2600 nm) is available. This laser system also generates the probe source for spectral encoding timing diagnostics employed to correct for instantaneous jitter and slow drift observed between the Aramis hard X-ray pulses and the experimental laser pump pulses at the interaction region.

#### 3.2.1. The experimental laser system

The experimental laser for the Alvra endstation comprises a Ti:sapphire-based system from Coherent Inc. A regenerative amplifier (Legend Elite) seeded by a Ti:sapphire oscillator (Vitara) is further amplified in two Ti:sapphire amplifier stages to provide upwards of 20 mJ pulses having a central wavelength of 800 nm, a bandwidth of 40 nm (FWHM), and a pulse duration of 35 fs (FWHM) upon optimal compression, with a repetition rate of 100 Hz. As 800 nm is not a wavelength broadly useful for initiation of most photochemical reactions, different options for conversion to other wavelengths are available at the endstation.

#### 3.2.2. Frequency conversion options from the fundamental

**Figure 4:**
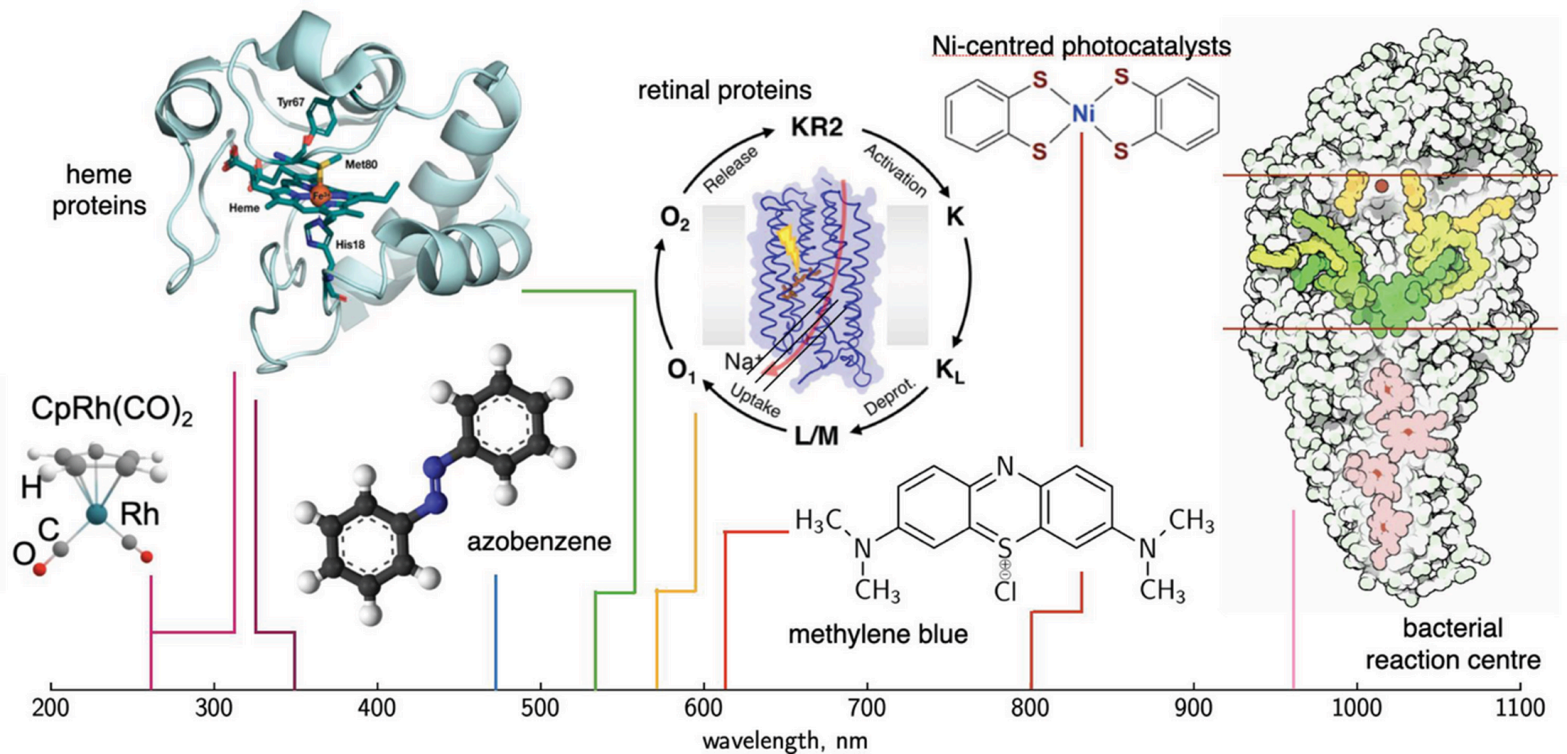
Representative selection of molecular and biological chromophores studied at the Alvra endstation at SwissFEL. Shown among others are heme and retinal proteins, organometallic complexes, metal centered photocatalysts and photosensitizers. The vertical lines indicate approximate absorption maxima, highlighting how the various chromophores exhibit light absorption and photo-activity across a broad range of wavelengths spanning from the ultraviolet to near-infrared spectral range.

Chromophores of all types are under study at the Alvra endstation, see Fig. 4 ▸, and as such multiple options for frequency conversion from the near-IR fundamental are needed depending on the pump pulse parameters of interest. For experiments where a direct harmonic of the fundamental is sufficient, second and third harmonic generation (to 400 nm and 266 nm, respectively) are readily available through nonlinear conversion of the fundamental in BBO crystals. More commonly, however, a commercial optical parametric amplifier (HE TOPAS; Light Conversion, UAB) is employed, offering tunable pulses with central wavelengths between 240 nm and 2.6 µm. These pulses are typically not compressed, and as a result of material dispersion through conventional optics [*e.g.* a variable optical density (OD) filter for attenuation, appropriate wave plates for polarization control, cut-off filters to remove residual fundamental light if needed, a lens for focusing the pump at the interaction region, and the window to enter the Alvra prime chamber] the pulse durations of the harmonics or the optical parametric amplifier (OPA) at the sample position are typically ≤100 fs FWHM. For the conventional focusing conditions in the Alvra Prime chamber (generally having FWHM diameters of 40–150 µm, depending on the experiment requirements), ≤10 µJ of pump photons are delivered to the interaction region which is readily available from either the harmonics or the OPA.

For scenarios where higher temporal resolution is needed, multiple options are under development based on spectral broadening of the fundamental (Xie *et al.*, 2021 ▸) or second harmonic (Xie *et al.*, 2024 ▸), or a home-built noncollinear optical parametric amplifier for ultrashort pulses tunable in the visible (Johnson *et al.*, 2011 ▸). These sources are compressed via chirped mirrors (specific for each wavelength range) to between 10 and 15 fs (FHWM) at the interaction region. Owing to the challenges associated with pulse compression and the high peak powers which result, these sources may not be suitable for all experiments and are available under special circumstances.

#### 3.2.3. Timing diagnostics

**Figure 5:**
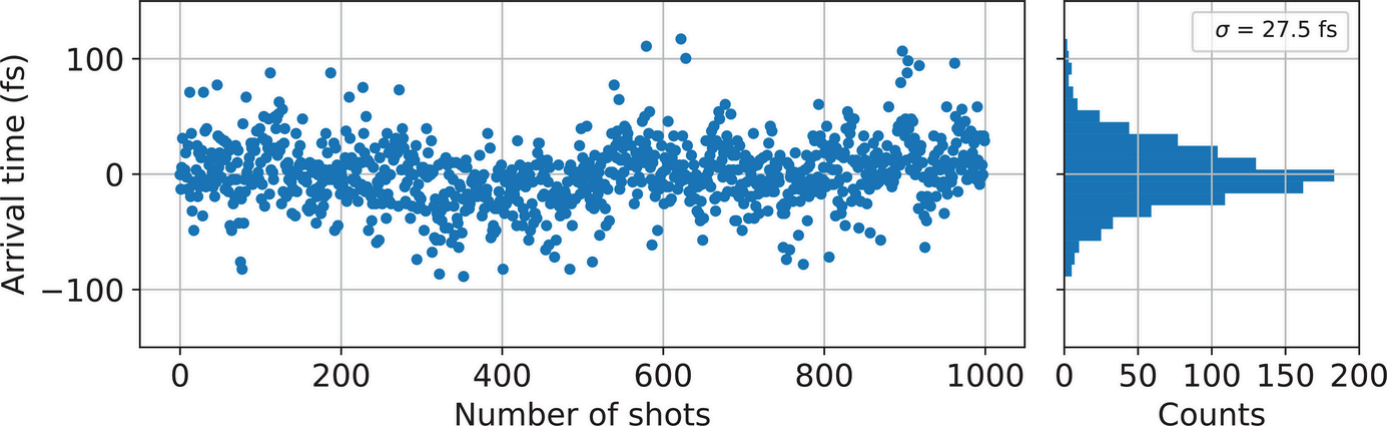
Arrival time jitter between the optical laser and 12 keV XFEL photon pulses as measured on a 20 µm YAG target by the downstream spectral encoder time tool. An ensemble of 1000 shots shows a normal distribution with standard deviation σ ≃ 30 fs.

All of the pulsed laser systems at SwissFEL are locked to a master oscillator reference via phase-locked loops. The master oscillator reference shows an integrated phase stability of ∼10 fs when measured between 10 Hz and 10 MHz. The slaved oscillators at the electron gun and experiment ends of the facility have ∼15 fs and ∼25 fs integrated phase noise, respectively. Owing to this intrinsic jitter of each of the pulsed laser sources at SwissFEL, and to the spontaneous nature of the SASE process to generate X-ray radiation in the undulator line of Aramis, the arrival time between the experimental laser and the X-rays has an intrinsic shot-to-shot uncertainty which must be measured independently to achieve the best possible temporal resolution. This is done either before and/or after the interaction region via spectral encoding (Harmand *et al.*, 2013 ▸). The dedicated spectral encoding devices consist of thin solid targets (typically 5 µm thick silicon nitride membranes, or 20–30 µm thick YAG crystals) which, upon excitation by the Aramis hard X-rays, exhibit a transient reflectivity change. A chirped white light supercontinuum pulse generated in a sapphire substrate acts as the arrival time probe, encoding in the transmitted spectrum the relative arrival time as a drop in intensity at a particular wavelength, as measured by a grating spectrometer. Through calibration of the temporal profile of the chirped supercontinuum probe, accurate arrival time information with ∼2 fs resolution is possible. This value represents the maximum accuracy with which arrival times can be inferred by the time tool and should not be interpreted as the overall temporal resolution achievable in an experiment. Fig. 5 ▸ shows a plot of the typical jitter between the optical laser and X-rays beams (12 keV pink X-ray photons, using a 20 µm YAG target in the downstream spectral encoding chamber), showing a normal distribution with a ∼30 fs r.m.s. deviation on 1000 shots.

Equally important to the instantaneous jitter between the X-rays and optical beam, slow drift arising due to atmospheric changes (predominantly through changes in ambient air pressure, but relative humidity and temperature can also play a role) results in non-trivial changes in the optical path length and index of refraction for the experimental laser pulses, leading to timing drifts on the order of 100 fs on hours-to-days timescales. To mitigate these effects, a feedback system monitors the output of the spectral encoding timing diagnostics and translates a mechanical delay stage on the fundamental beam, keeping the arrival time fixed within the instantaneous jitter window. As a low repetition rate XFEL, photon-hungry experimental methods at SwissFEL require long acquisition times to achieve sufficient SNR, and thus the use of feedback on the arrival time information from the timing diagnostics is crucial to realize high temporal resolution of the ultrafast processes of interest.

The Alvra beamline is equipped with two spectral encoder time tools, located before and after the Prime experimental station, see Fig. 3 ▸. Depending on the constraints of the experimental setup, either the upstream, the downstream or both devices are inserted into the beam path and put in operation. It has been demonstrated that they can provide signal levels good enough to reliably correct slow drifts and extract instantaneous jitters over the entire photon energy range of the Aramis branch, either with the pink or monochromatic beam.

### 3.3. Techniques and data examples

In this section we will present some of the experimental results obtained at the Alvra endstation during the early years of SwissFEL’s commissioning and operation. We will limit the discussion to the endstation capabilities for liquid sample delivery, employing time-resolved X-ray spectroscopy (XAS and XES, RIXS) and X-ray solution scattering (XSS). Serial femtosecond crystallography, despite also being a commonly used technique at Alvra (Skopintsev *et al.*, 2020 ▸; Mous *et al.*, 2022 ▸; Wranik *et al.*, 2023 ▸; Gruhl *et al.*, 2023 ▸; Christou *et al.*, 2023 ▸; Cellini *et al.*, 2024 ▸; Barends *et al.*, 2024 ▸), is beyond the scope of this paper and will be described separately in a future publication.

#### 3.3.1. X-ray absorption spectroscopy

XAS is an element-specific and local bonding-sensitive technique that determines the partial density of the unoccupied states of the target system (Yano & Yachandra, 2009 ▸). As such, XAS has become one of the most fundamental techniques used to measure atomic-scale structures with angstrom precision, even in amorphous media like liquids.

XANES refers to the region of the XAS spectrum near an element’s absorption edge, where the largest variations in the X-ray absorption coefficient and consequently intense, narrow resonances are found. When performed in a time-resolved manner, tr-XANES gives insight into the dynamical changes of both structural and electronic configurations in various chemical and biological systems like solvated ions, active centers in proteins and metal based molecular species (Gawelda *et al.*, 2007 ▸; Chen *et al.*, 2007 ▸; Lemke *et al.*, 2013 ▸; Bacellar *et al.*, 2020 ▸).

The Fe metal center complex [Fe(1,10-phenanthroline)_3_]^2+^ [Fe^2+^(phen)_3_] belongs to the last category and is known to be one of the systems that exhibits an SCO mechanism (Kabanova *et al.*, 2024 ▸). SCO is a phenomenon for which the spin state of the metal complex changes due to an external excitation, which could be provided by a change of temperature, pressure or induced by a laser pulse. As reported extensively in the literature (Hauser, 2004 ▸; Bressler *et al.*, 2009 ▸; Cannizzo *et al.*, 2010 ▸; Auböck & Chergui, 2015 ▸; Lemke *et al.*, 2017 ▸), the photoexcitation of such systems induces the population of an electronic metal-to-ligand charge transfer (MLCT) state, which decays to an HS state within a few hundred femtoseconds, from which the system relaxes back to the ground state after several hundreds of picoseconds. The population of the HS state is accompanied by the formation of a coherent vibrational wave packet, which is encoded in the transient XANES spectra as a series of damped oscillations with a characteristic period of hundreds of femtoseconds.

We performed a pump–probe experiment using ultrashort optical laser pulses as the pump and X-ray pulses as the probe. The former pulses were generated by the home-built non-collinear optical parametric amplifier (NOPA) with a pulse duration of approximately 8 fs (FWHM), measured with a transient-grating frequency-resolved optical gating (TG-FROG) setup (Sweetser *et al.*, 1997 ▸). The X-rays were produced upstream of the undulators by tilting the electron beam using a passive wakefield device (Lutman *et al.*, 2016 ▸) and characterized downstream of the undulators (Dijkstal *et al.*, 2022 ▸). The estimated X-ray pulse duration was 12 fs (FWHM) and pulse energies of approximately 500 µJ per pulse. Under these conditions, we collected the time-resolved XANES signal of a 20 m*M* aqueous solution of Fe^2+^(phen)_3_ delivered to the interaction point with a 25 µm diameter cylindrical liquid jet. The X-ray spot size was approximately 25 µm × 25 µm (FWHM), while the optical laser measured 38 µm × 38 µm (FWHM). The optical excitation spectrum was centered around 532 nm, close to the absorption peak of the MLCT band, and the pulse energy was set to 0.5 µJ per pulse, which was measured to be below the onset of a nonlinear signal response. The overall instrument response function (IRF), determined with a cross-correlation measurement on a 2 µm thick SiN target, was approximately 35 fs FWHM, which was sufficient to resolve the ultrafast response of the sample.

Two large area silicon avalanche photodiodes (APDs) with dimensions of 10 mm × 10 mm (Excelitas Technologies C30703FH-200) were used to collect the emitted X-rays in total fluorescence yield (TFY) mode. They were placed approximately 10 mm away on both sides of the liquid jet at 90° with respect to the incoming X-ray beam in order to optimize the collection efficiency of fluorescence radiation whilst minimizing the contribution of elastically scattered photons. The active surface of both detectors was shielded with one foil of Mn (6 µm thick) acting as *Z*−1 filter and another one of Al (6 µm thick). Using these filters, the detection of elastic scattering radiation from the jet can be further minimized, whilst most of the fluorescence from the Fe atom is preserved. Furthermore, the presence of metal filters inhibits the spurious detection of the pump visible light at the cost of a slightly reduced transmitted fluorescence. The applied biases to the detectors were tuned to −310 V and −290 V, respectively, such that the APDs were working in the optimal linear gain regime, whilst avoiding saturation. The signals were then processed by an analog-to-digital converter (ADC, Keysight) where the waveforms were integrated and the backgrounds subtracted on a single-shot basis, returning a scalar value for the non-normalized single-shot detected fluorescence, *F*_0_.

Because of the energy jitter of the SwissFEL accelerator and the stochastic intensity variations of the SASE-based XFEL pulses, the shot-to-shot intensity fluctuations of the monochromatic beam are usually of the order of 100%. Such large intensity fluctuations of the monochromatic beam require the normalization of the single-shot fluorescence yield *F* = *F*_0_/*I*_0_, where *I*_0_ is the intensity of the XFEL pulse, measured with one of the non-destructive intensity monitors (PBPS) positioned after the DCM.

**Figure 6:**
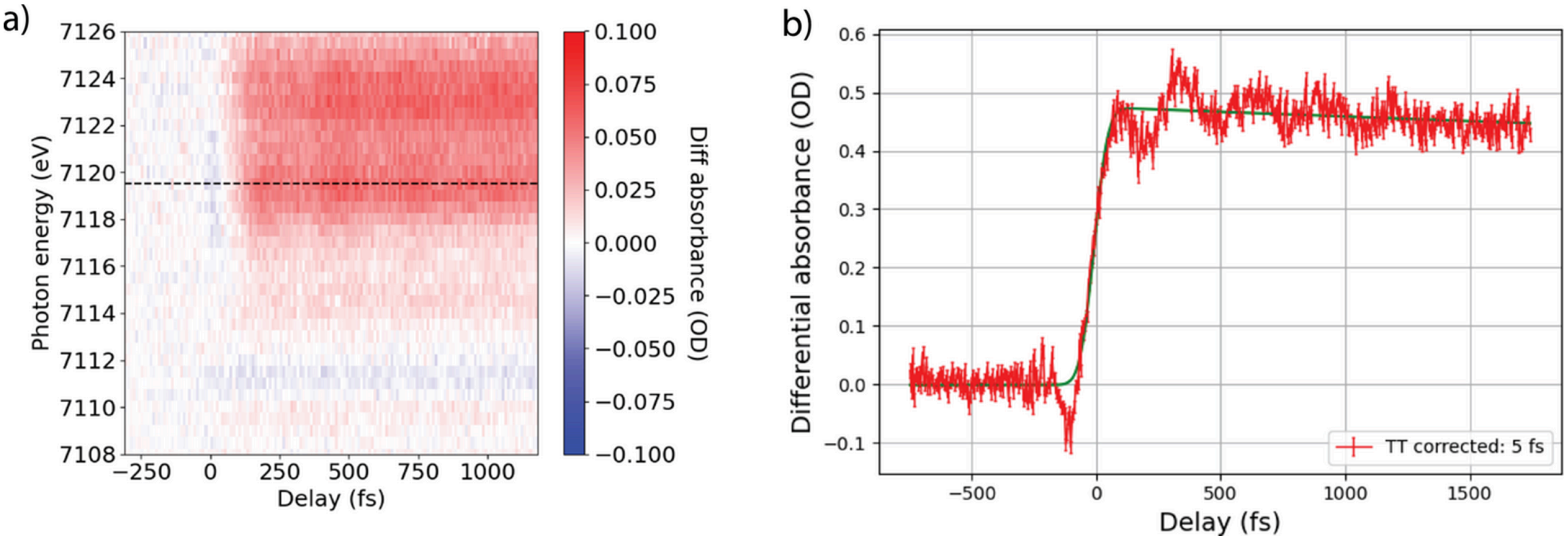
Time-resolved XANES measurement of aqueous Fe^2+^(phen)_3_. Panel (*a*) shows the differential absorbance measured across the Fe *K*-edge (7108 to 7126 eV in 0.5 eV step size) for relative delays between the laser pump and the X-ray probe in the range −500 to 1000 fs. Panel (*b*) shows a time scan of the relative absorption change at an X-ray energy of 7119.5 eV, marked as a dashed line in panel (*a*). The fit (green solid line) returns a sample response of about 110 fs, in agreement with an LS–HS transition measured for similar systems (Lemke *et al.*, 2017 ▸).

Figs. 6 ▸ and 7 ▸ show the results of a XANES measurement performed at the Alvra endstation on a sample of Fe^2+^(phen)_3_. The photon energy axis was calibrated using the DCM readout rather than a reference sample. The DCM calibration was carried out across the full Aramis energy range by measuring the transmission through a series of metal filters.

The data acquisition was performed with SwissFEL running at the standard repetition rate of 100 Hz. Every second pump laser was blocked to generate a sequence of alternating ‘on’ (both laser and XFEL pulses reaching the sample) and ‘off’ (only XFEL pulses reaching the sample) shots. The odd–even ordering of this sequence was periodically swapped to prevent the pumped shots from being always coupled to the FEL pulses of the same parity. This approach minimizes systematic errors associated with the fundamental frequency of the power grid. For each pair of fluorescence signals with and without laser, *F*_0,on_ and *F*_0,off_, respectively, the normalized fluorescence signals *F*_on_ and *F*_off_ were calculated using the correlated *I*_0_ of the XFEL pulse: *F*_on_ = *F*_0,on_/*I*_0,on_ and *F*_off_ = *F*_0,off_/*I*_0,off_.

The diode positions, shielding and biases were optimized to obtain the highest Pearson correlation coefficient, *R*, between the fluorescence *F*_0_ and the XFEL intensity *I*_0_. The differential absorbance was calculated as Δ*A* = 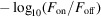 after filtering for the most well correlated shots. For the data shown in Fig. 6 ▸ values of *R* > 0.99 were obtained for both subsets of *F*_on_ and *F*_off_. Shots within the 70th percentile were averaged to calculate Δ*A*.

The transient absorption spectra shown in Fig. 6 ▸(*a*) were collected tuning the DCM to a set of 37 energies distributed between 7108 and 7126 eV in steps of 0.5 eV. For any specific energy set on the DCM, the Aramis undulator gaps were also varied in order to be able to scan an energy range larger than the SASE XFEL spectrum and achieve a uniform X-ray flux reaching the sample across the whole photon energy range. At the same time, the optical laser delay stage was run continuously with a speed of about 30–40 µm s^−1^ (corresponding to ∼2 fs per shot at 100 Hz), between two set points corresponding to a nominal delay between pump and probe of between −500 fs (pump *after* FEL) and 1000 fs (pump *before* FEL). The stage position was tagged for each shot and the final delay axis of Fig. 6 ▸ was reconstructed by adjusting the delay value with the arrival time information provided by the time tool positioned on the back of the Prime chamber. The transient XANES data are generated by averaging ten repetitions of the 2D scans, recorded in a total time of 6 h, resulting in a total of approximately 1.75 million on/off pairs. The single-shot data are distributed along the time axis according to the readout of the delay stage encoder position corrected by the time tool arrival time. Data belonging to the same delay bin of time width equal to 10 fs were subsequently averaged.

The kinetic trace shown in Fig. 6 ▸(*b*) is the average of three scans measured with the DCM aligned at an energy of 7119.5 eV. For each scan, the delay stage was moved between −750 and 2000 fs in 25 fs steps (110 steps) and 1000 shots per step were collected, with the same alternating ‘on/off’ illumination scheme as used above. The data shown in Fig. 6 ▸(*b*) were collected in about 70 min of continuous data collection. A fit of the rise time of the XANES kinetic trace [green solid trace in Fig. 6 ▸(*b*)] returns a value of approximately 110 fs, which is consistent with the LS–HS transition measured for similar systems (Lemke *et al.*, 2017 ▸).

**Figure 7:**
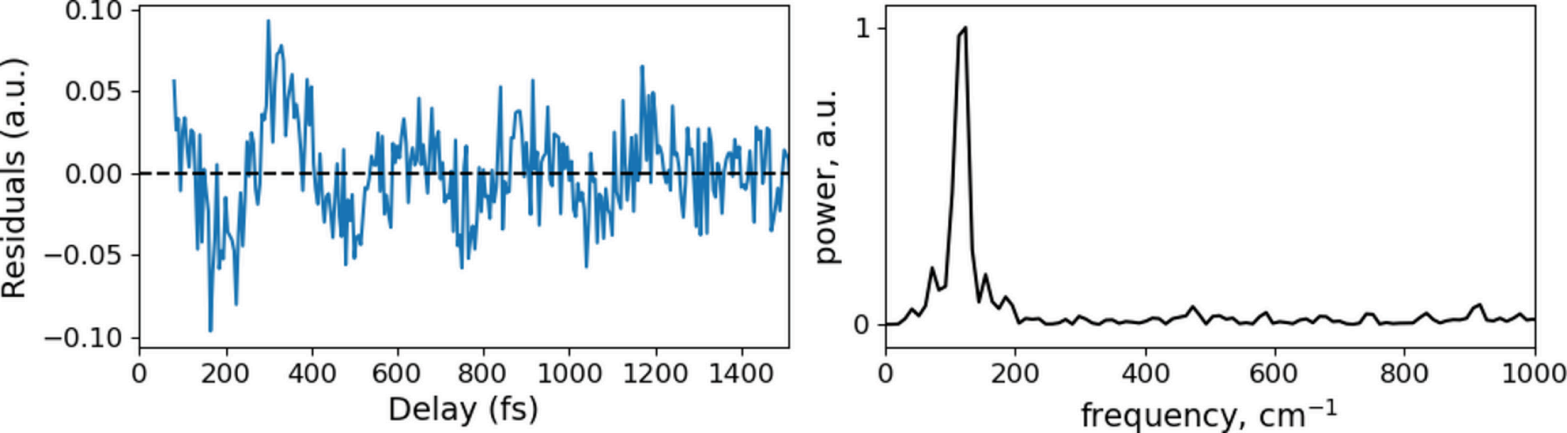
The left panel shows the residuals between the fit and the experimental data shown in Fig. 6 ▸(*b*). The right panel presents the power spectrum of the residuals calculated via fast Fourier transform (FFT), revealing damped oscillations at 120 cm^−1^ (280 fs), in agreement with previously reported values for similar systems (Lemke *et al.*, 2017 ▸).

In the transient absorption plot of Fig. 6 ▸(*a*), for photon energies between 7118 and 7122 eV, it is possible to distinguish the negative feature attributed to the excitation of the system into the MLCT state. This is followed by oscillations representing the coherent vibrational wave packet dynamics in the HS state, with the characteristic period of ∼280 fs (120 cm^−1^) (see also Fig. 7 ▸). The same features are observed in the kinetic trace measured at a single energy displayed in Fig. 6 ▸(*b*). A subtraction of the fit curve from the data returns the residuals shown on the left side of Fig. 7 ▸. The power spectrum of these residuals data reveals the period of the coherent oscillations which are attributed to the *a*_1*g*_ ligand breathing mode in-phase with the Fe—N bond stretching (Lemke *et al.*, 2017 ▸).

#### 3.3.2. X-ray emission spectroscopy

XES is an element-specific method to probe the partially occupied electronic structure of materials. XES is a complementary technique to XAS and provides information on the electronic structure, in particular local charge and spin density. In recent years, time-resolved XES has developed as one of the most useful tools to probe both the electronic structural changes and dynamics of molecules and materials (Urch, 1971 ▸; Meisel *et al.*, 1989 ▸; De Groot, 2001 ▸; Bhargava *et al.*, 2018 ▸).

XES employs an analyzer crystal to disperse scattered or fluorescence X-ray photons on a pixel detector a high energy resolution limited by the core-hole lifetime of the system under investigation. For measurements with XFEL radiation, the incident photon energy can be tuned either off-resonance (non-resonant XES, generally performed employing the entire XFEL SASE spectrum) or on-resonance with an absorption edge (resonant XES, also known as RIXS, typically achieved using a monochromatic beam). In the current section we show data for time-resolved non-resonant XES, while in Section 3.3.3 we present time-resolved RIXS data measured with tender X-ray incident radiation (photon energy ≃ 3000 eV).

**Figure 8:**
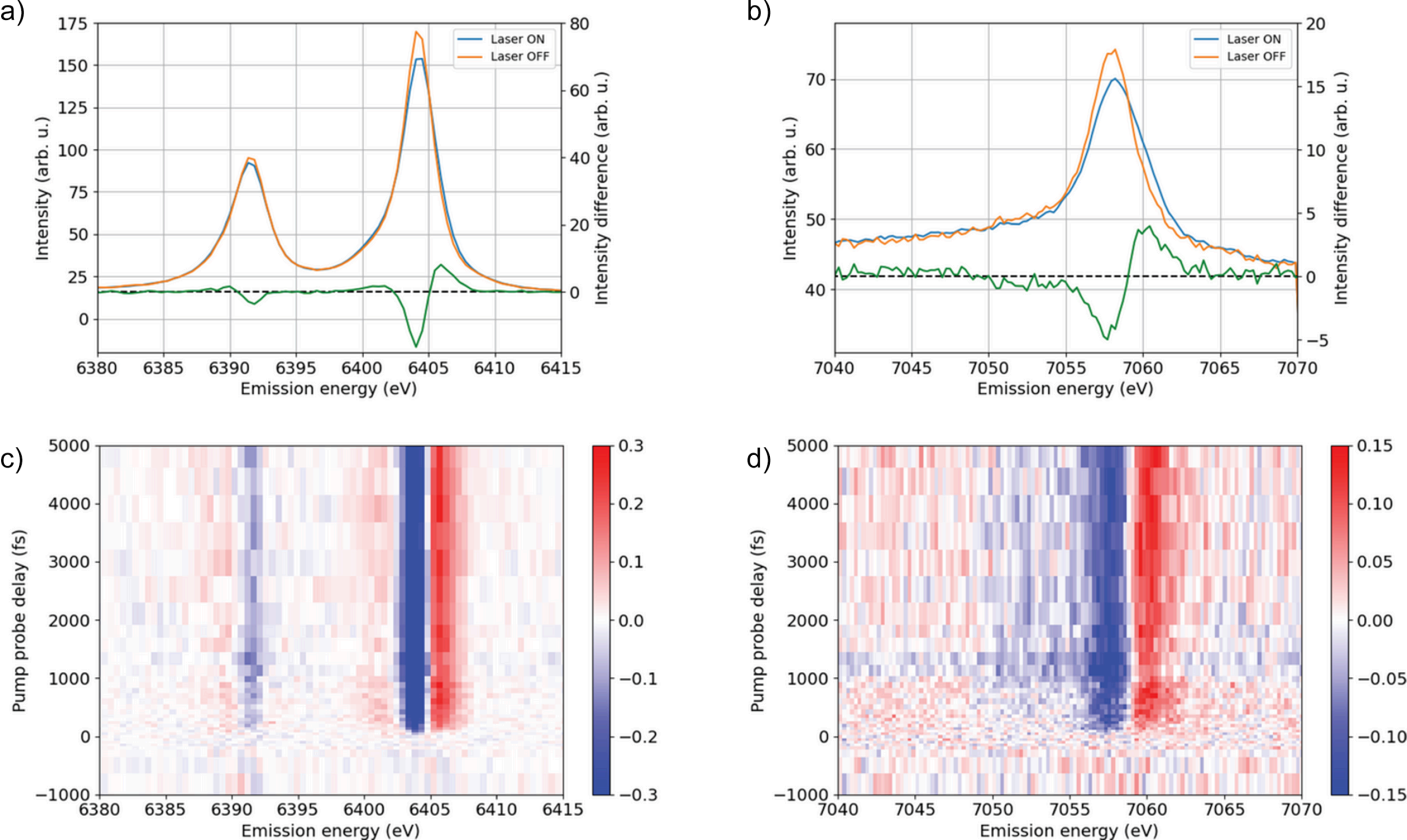
Averaged spectra of the Fe *K*_α1,2_ (*a*) and *K*_β_ (*b*) lines for a 10 m*M* aqueous solution of Fe^2+^(phen)_3_. Spectra with laser are shown as blue solid lines, without laser as orange solid lines, and their difference measured at 5 ps delay as green solid lines. Time-resolved two-dimensional *K*_α1,2_ (*c*) and *K*_β_ (*d*) fluorescence difference spectra for delay values between −1 and +5 ps.

Non-resonant time-resolved X-ray emission (tr-XES) data collected on an aqueous solution of Fe^2+^(phen)_3_ are shown in Fig. 8 ▸. The 10 m*M* solution was delivered to the interaction region with a 100 µm diameter cylindrical liquid jet. X-ray fluorescence photons from the valence-to-core Rh transition (2*p*4*d* Rh *L*_β1_ and Rh *L*_β2, 15_ emission lines) generated by the absorption of the SASE pink beam centered at 11 keV (0.2% bandwidth) were collected using a von Hamos X-ray emission spectrometer installed in Prime. The spectrometer employed two pairs of short focal length (radius of curvature of 250 mm) cylindrically bent segmented crystals aligned to simultaneously collect radiation from Fe 2*p*–1*sK*_α_ [crystal cut Si(331), Bragg angle ≃ 51°] and Fe 3*p*–1*sK*_β_ [crystal cut Si(531), Bragg angle ≃ 73°] emission lines and focus them onto the 4.5M JUNGFRAU detector.

The sample was excited with ∼70 fs pump laser pulses with a central wavelength of 535 nm, close to the absorption peak of the MLCT band. The excitation pulse energy was set to 4 µJ per pulse, as was measured to be below the onset of any nonlinear effects.

For each collected single-shot JUNGFRAU image, we performed a pixel dependent dark current (pedestal) subtraction and a gain correction provided by a calibrated map. Due to the low efficiency of the von Hamos spectrometer and the consequent low photon yield, all the pixels of the JUNGFRAU detector were operating in the high gain mode. High and low intensity thresholds were applied to reduce the noise originating both from inelastic scattering directly hitting the detector without fulfilling the Bragg scattering geometry and from hot pixel readings. After careful optimization, values of 3 keV and 15 keV were chosen for the low and high threshold values, respectively. The low threshold value was set to be low enough to preserve counts from rare events for which a *K*_α_ XES photon falls in between two adjacent pixels, while the high threshold value imposed a limit for a maximum detection of two photons per pixel per shot.

Regions of interest around the Fe *K*_α_ and *K*_β_ emission lines were cropped from the 2D calibrated images and an integration along the non-dispersive axis returned the fluorescence lines emission spectra, shown in Figs. 8 ▸(*a*) and 8 ▸(*b*). During the measurement, fluorescence images were collected continuously following a regime of six pumped shots followed by one un-pumped shot, with the dark shot coupled alternately to odd and even XFEL pulses, thus mitigating systematic errors associated with the power grid’s fundamental frequency. The time scans shown in Figs. 8 ▸(*c*) and 8 ▸(*d*) were performed varying the pump–probe delay in steps between −1 and 5 ps, recording 2000 shots per step. The 2D *K*_α_ and *K*_β_ fluorescence difference spectra are the result of 19 averaged time scans, collected over approximately 4.5 h, with the delay axis corrected and reconstructed using the time tool arrival time information. The emission energy axis was calibrated by comparing the measured emission line shapes with tabulated ones from reference samples.

**Figure 9:**
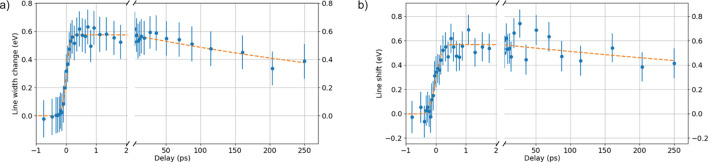
Panel (*a*) shows the *K*_α1_ line width extracted from an asymmetric Voigt fit at 6405 keV. Panel (*b*) shows the *K*_β_ line shift derived from an asymmetric Voigt fit at 7059 keV. The orange dashed traces represent the fits to the data, obtained using a function with a rise limited by the IRF and a bi-exponential decay. The fits return time constants of about 600 and 800 ps for *K*_α1_ and *K*_β_, respectively.

Fig. 9 ▸ shows the laser induced effect on the *K*_α1_ and *K*_β_ line shapes. The experimental data (blue solid circles with error bars) are extracted from the fit of the *K*_α1_ (*a*) and *K*_β_ (*b*) XES data, performed using an asymmetric pseudo-Voigt function as described by Bacellar *et al.* (2020 ▸). The line width change and peak shift, for *K*_α1_ and *K*_β_, respectively, returned by the fit are plotted as a function of the pump–probe delay after averaging all the pumped shots. To validate this method, we verified that no change in the line width and peak position is observed if the averaged dark shots (without laser excitation) are analyzed. The dashed orange lines are the fits to the data obtained by a bi-exponential decay function with an IRF limited rise time.

As for the XANES presented previously, the total temporal resolution of the experiment results from the convolution of the optical and X-ray pulse durations, the group velocity mismatch between the X-ray and optical pulses in the sample and the error in the arrival time measurement from the time tool. For the presented data, the convolution of these terms would predict an IRF of approximately 120 fs, which is in agreement with the width of a cross-correlation measurement run on a solid YAG target. In the data analysis of the emission data from Fe^2+^(phen)_3_, the instrumental response function and time zero (coincident arrival of the X-ray and optical pulses) are left as fit parameters.

The rise time extracted from the *K*_β_ kinetic traces is 330 ± 40 fs, while for *K*_α1_ it is 175 ± 25 fs. Although on a similar time scale, both values are larger than the IRF determined separately, suggesting a contribution of an intermediate excited state. Furthermore, the slower rise time measured on the *K*_β_ line with respect to *K*_α1_ may be due to competing intersystem crossing and structural dynamics, as suggested by Canton *et al.* (2023 ▸). The XES kinetic traces demonstrate that efficient LS to HS state switching takes place on a sub-picosecond timescale. The recovery to LS (ground) state happens on a much longer timescale. The exponential fits applied to both *K*_α1_ and *K*_β_ kinetic data show a long-lived excited state, the HS, with decay constants in the order of 700 ps, in agreement with the value reported in the literature (Kabanova *et al.*, 2024 ▸).

#### 3.3.3. Tender X-ray spectroscopy

The tender X-ray range, generally referring to photons with an energy between 2 and 5 keV, is of particular interest at the Aramis branch. SwissFEL is among the first XFELs capable of reaching this energy range. While XAS measurements in the tender range can also be performed at the South Korean XFEL facility PAL-XFEL (Kim *et al.*, 2022 ▸), to date Alvra remains the only endstation worldwide equipped with an emission spectrometer that permits detection of tender X-ray emission, where *K*-edges of light elements as well as *L*-edges of 4*d* transition metals lie. As such, Alvra is the only endstation that can perform RIXS or simultaneous XAS and XES experiments in the tender X-ray photon energy range (Banerjee *et al.*, 2024 ▸; Jay *et al.*, 2023 ▸; Lim *et al.*, 2025 ▸; Garratt *et al.*, 2025 ▸).

**Figure 10:**
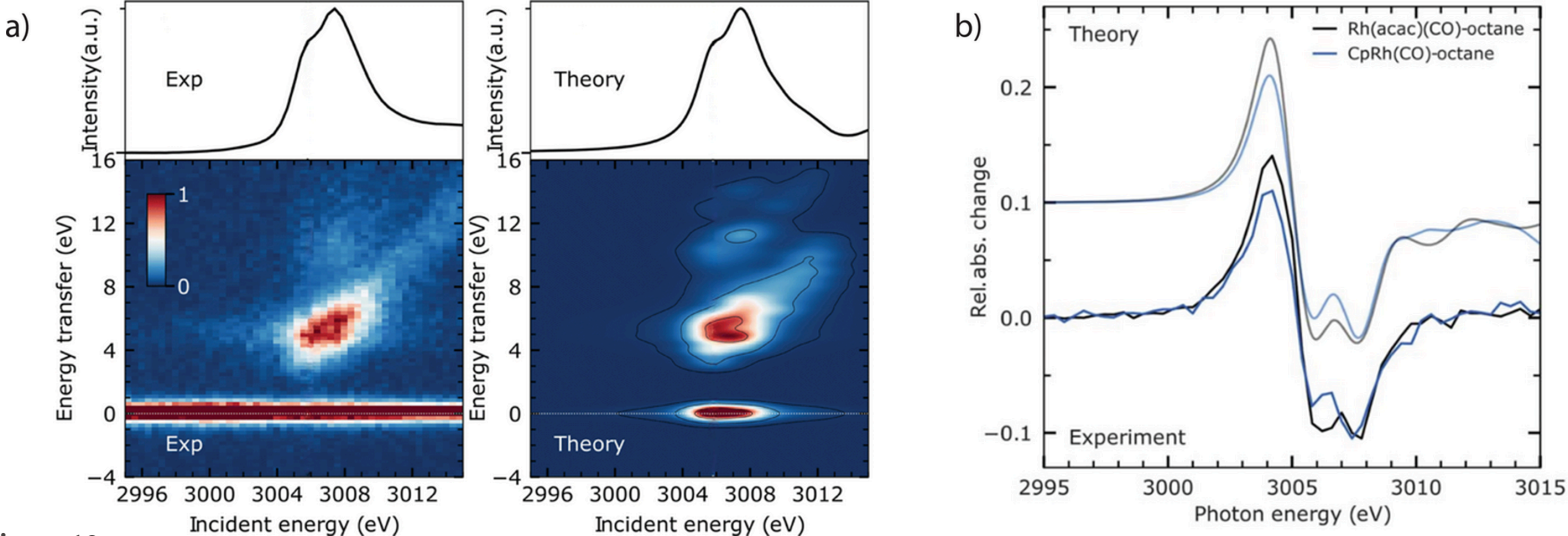
(*a*) Comparison between measured (left panel) and calculated (right panel) 2D steady-state VtC-RIXS plane measured on a 20 m*M* concentrated solution of CpRh(CO)_2_ dissolved in decane. (*b*) Transient difference XANES of CpRh(CO)_2_ and Rh(acac)_2_ dissolved in octane, measured at the Rh *L*_3_-edge at a pump–probe delay of 10 ps. The measured data for both samples (dark blue and black) are compared with calculated traces (light blue and gray). Adapted from Banerjee *et al.* (2024 ▸) and from Jay *et al.* (2023 ▸).

Fig. 10 ▸ shows RIXS and XANES spectra acquired for a cyclopentadienyl rhodium carbonyl complex CpRh(CO)_2_ diluted in decane and octane, respectively, probed at the Rh *L*_3_-edge, located at a photon energy of ∼3005 eV (Banerjee *et al.*, 2024 ▸; Jay *et al.*, 2023 ▸).

The 20 m*M* solution was delivered to the interaction point with a 75 µm diameter cylindrical liquid jet. After a series of air-purging and He-refilling cycles, the Prime chamber was kept at 500 mbar He atmosphere to minimize X-ray scattering and increase transmission. Using the Si(111) crystals in the Alvra DCM, the photon energy was tuned across the Rh *L*_3_-edge, between 2995 eV and 3015 eV. Time-resolved RIXS and XANES data were collected by optically exciting the sample with a 266 nm laser pulse and then probing the sample with X-rays. The laser was blocked every second pulse, giving consecutive laser-on/laser-off shots. The X-ray fluorescence emerging from the sample was collected with an APD placed on the right side of the liquid jet at an angle of 90° with respect to the incident X-ray beam. The X-ray fluorescence was simultaneously dispersed by a pair of short focal length (radius of curvature of 250 mm) cylindrically bent Si(111) segmented crystals installed in the von Hamos XES spectrometer located on the left side of the liquid jet. Fig. 10 ▸(*a*) presents the RIXS data as two-dimensional (2D) plots of incident energy versus energy transfer. Note that these 2D plots show steady-state data (*i.e.* data from laser-off shots only).

The elastic scattering peak position and width were used to calibrate the energy axis of the von Hamos spectrometer and to estimate its energy resolution, respectively. The latter was determined to be ∼0.8 eV at an incident energy of 2995 eV.

The data treatment was previously presented in Section 3.3.2 for the XES data of Fe^2+^(phen)_3_. The single-shot JUNGFRAU images were corrected by subtracting a background (pedestal) and applying a pixel-based gain map. Subsequently, the measured pixel signals were converted to photon energy in units of keV. Low and high energy thresholds (2.0 and 10.0 keV, respectively) were applied to each pixel to minimize noise and eliminate hot pixels counts.

The 2D RIXS plot shown in Fig. 10 ▸(*a*) was obtained by averaging 24 energy scans, each of them containing 51 energy values equally distributed between 2995 and 3015 eV. For each energy step, 350 laser-off shots were recorded. The total number of shots used to generate the 2D RIXS plot was therefore approximately 425000 and was acquired in about 2.5 h. For the XANES data of Fig. 10 ▸(*b*), we adopted the same acquisition scheme as for the RIXS data. The dataset of CpRh(CO)_2_ [light blue line in Fig. 10 ▸(*b*)] is the result of an average of nine consecutive scans, for a total of ∼160000 shots. This dataset was recorded in about 55 min. Fig. 10 ▸(*b*) shows also the differential spectrum for another complex, namely Rh(acac)(CO)_2_ dissolved in octane (dark blue line). Due to the higher absorption coefficient and photolysis yield, the spectra for Rh(acac)(CO)_2_ required the acquisition of fewer shots than CpRh(CO)_2_ to reach the same signal-to-noise ratio. Only two scans were averaged for the dataset shown in Fig. 10 ▸(*b*), which was recorded in approximately 12 min.

#### 3.3.4. X-ray solution scattering

XSS is an elastic scattering technique that involves the interaction of X-rays with a sample in a liquid state (Ihee *et al.*, 2010 ▸; Borfecchia *et al.*, 2013 ▸; Kjær *et al.*, 2019 ▸). The analysis of the scattered X-rays can reveal information about the size, shape and arrangement of the solute molecules, as well as details of their interactions with the solvent molecules.

Time-resolved XSS captures the dynamics of molecules and their structural changes in real time. This method is particularly useful for studying rapid processes, such as protein folding, conformational changes and biochemical reactions. A short optical laser pulse (pump) excites the sample under study, while a subsequent X-ray pulse probes the dynamics at different time delays between the pump and the probe. The pump beam excites only a small portion of the sample and it is the difference in the scattered intensity as a function of scattering angle for data collected from sample with and without the laser excitation that contains the relevant signal. This differential intensity can be decomposed into three components: the solute term, the solvent term and a cross term (encompassing solute–solvent interactions, also referred to as the ‘cage term’) (Borfecchia *et al.*, 2013 ▸; Ihee *et al.*, 2010 ▸; Kong *et al.*, 2010 ▸; Kjær *et al.*, 2019 ▸; Cammarata *et al.*, 2006 ▸; Haldrup *et al.*, 2010 ▸),
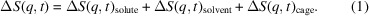
The detected scattering signals are sensitive to all the species present in the solute and solvent. Usually, the information is extracted by comparing the experimental data with signals obtained by scattering models calculated using the 3D atomic coordinates for the ground and excited states of the chemical species involved. The Δ*S*(*q*, *t*)_solvent_ term has to be reliably removed to obtain the true signal from the solute; however, since the solvent signal is often dominant, this correction has to be made carefully. The solvent term is very sensitive to the thermodynamic properties of the solvent, such as temperature, density and pressure, which change during and after laser excitation due to energy transfer from the solute to the solvent molecules. Generally, the solvent response can be described as a linear combination of two independent thermodynamical parameters like temperature *T* and density ρ,
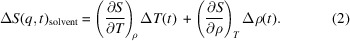
It can be shown that after fast vibrational cooling of the initially excited molecules – which happens within the first few tens of picoseconds – the deposited energy is transferred to the bulk solvent, and the solvent response can be described purely in terms of hydrodynamic variables such as temperature, pressure and density (Kjær *et al.*, 2013 ▸). Therefore, the partial derivatives of equation (2) can be obtained by measuring data at ‘late’ time delays. In particular, when the X-ray probe is delayed by 100 ps relative to the laser excitation pump pulse, the scattering data correspond essentially to 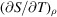 multiplied by the early stage of heating happening at constant volume. Thus, the contribution of the second term of equation (2) can be neglected. The second term 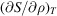 becomes relevant at even later time delays and can be retrieved by measuring scattering data collected 1 µs after laser excitation, when the solvent is still hot but its temperature has lowered from the ‘equilibrium’ temperature reached at 100 ps due to expansion. Once the solvent heat term is calculated with this procedure, the remaining contributions of the solute and solute–solvent terms can be extracted from the time-resolved XSS (tr-XSS) measurements.

In tr-XSS experiments performed at the Alvra Prime endstation, the sample is delivered via a cylindrical liquid jet and the scattering images are recorded on the JUNGFRAU 16M detector positioned approximately 10 cm downstream from the sample–X-ray interaction region. The tr-XSS data are collected in a sequence with several consecutive ‘light’ shots (with sample excited by pump laser pulses) interleaved by one ‘dark’ shot (without pump laser). The standard illumination scheme employed at Alvra alternates one dark shot after six light shots, such that the dark shot is alternately coupled to odd and even XFEL pulses. This helps to mitigate systematic errors coming from the fundamental frequency of the power grid.

After calibrating the detector geometry using diffraction data from LaB_6_ powder as reference sample, the scattering images are azimuthally integrated to return the 1D scattering signal *S*(*q*, *t*). A running average of six dark images *S*_laser-off_ is subtracted from each light image *S*_laser-on_ such that a single-shot differential signal Δ*S*(*q*, *t*) = *S*(*q*, *t*)_laser-on_ − *S*(*q*, *t*)_laser-off_ can be calculated.

**Figure 11:**
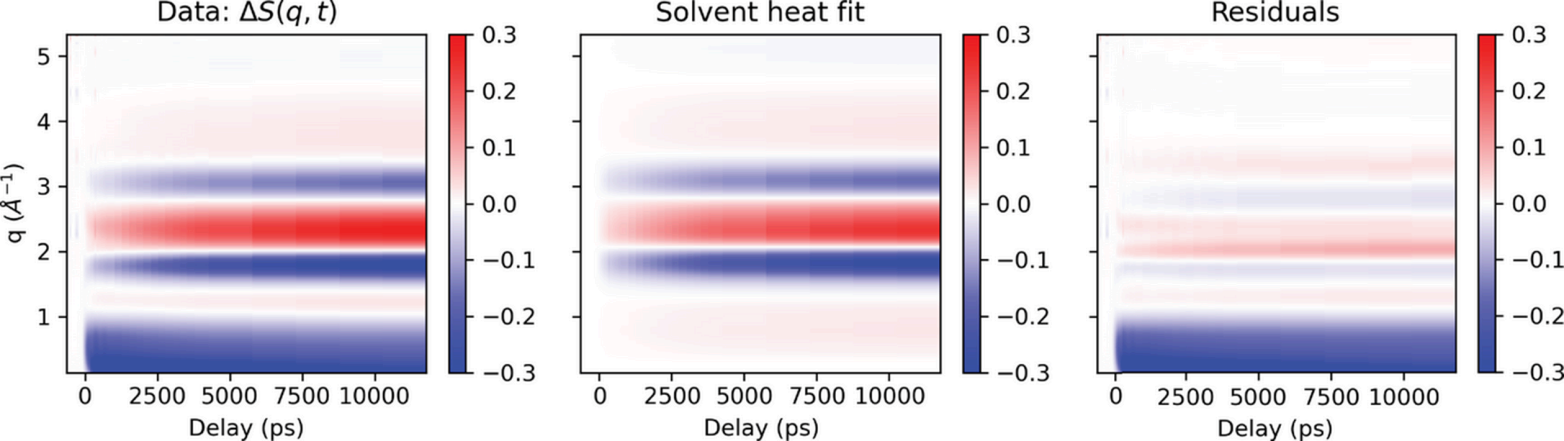
Time-resolved difference XSS signals generated by 10 m*M* solution of Fe^2+^(phen)_3_ in water photoexcited at 400 nm. The panel on the left shows the measured difference scattering signal Δ*S*(*q*, *t*) as a function of pump–probe delay *t*. The central panel shows the results of the fit run considering only the solvent heating term Δ*S*(*q*, *t*)_solvent_ presented in equation (2) of the text. The right-hand panel shows the residuals obtained by subtracting the solvent heating contribution from the experimental data.

An example of such a measurement is shown in Fig. 11 ▸(*a*). A 10 m*M* solution of Fe^2+^(phen)_3_ was delivered to the interaction region through a 100 µm diameter cylindrical liquid jet. The sample was excited by ∼30 fs pump laser pulses at a wavelength of 400 nm, with a pulse energy of 5 µJ per pulse and spot size of ∼35 µm × 35 µm. The system dynamics were probed using 8.7 keV SASE XFEL pulses with pulse energies of approximately 800 µJ per pulse. The 2D plot represented here was recorded over approximately 3.5 h and is the result of an average of 15 consecutive scans, taken from a total of ∼980k shots distributed over 46 delay values unevenly spaced between −400 fs and 12.5 ps.

As already introduced in the previous sections, the dynamics of aqueous Fe^2+^(phen)_3_ can be described as an excitation of the system from the singlet ground state into an MLCT excited state that decays rapidly (<1 ps) into a very long lived metal-centered quintet excited state (^5^*T*_2_).

**Figure 12:**
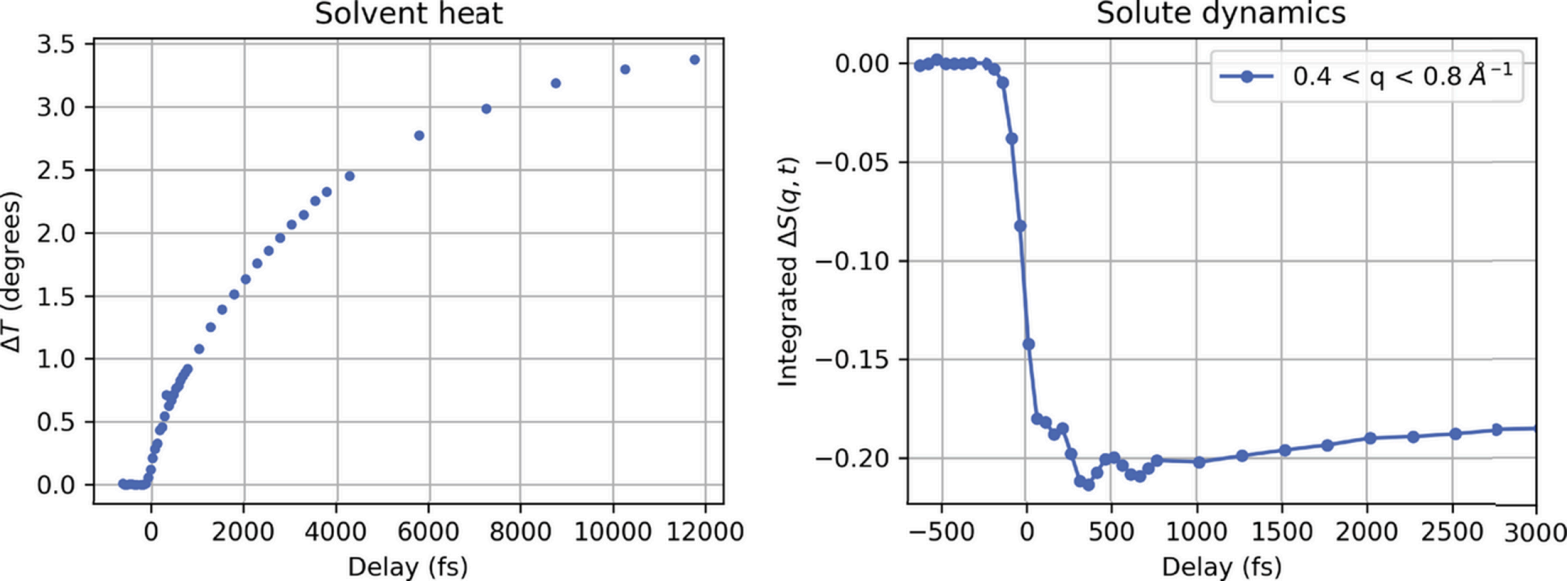
Solvent and solute responses extracted from the time-resolved XSS measurements shown in Fig. 11 ▸. The left panel shows the solvent temperature rise as retrieved from the fit. The right panel shows the time-resolved signal for low *q* range, integrated between 0.4 and 0.8 Å^−1^. For time delays shorter than 1 ps, the integrated data show coherent oscillations with a period of ∼300 fs, in agreement with the XANES data shown in Figs. 6 ▸ and 7 ▸.

The tr-XSS data presented in Fig. 11 ▸ can be used to confirm these results and complement them with additional information about the solvent and solute–solvent dynamics. A large contribution to the data shown in Fig. 11 ▸(*a*) is given by the solvent heating, which can calculated using equation (2). As described above, the term (∂*S*/∂ρ)_*T*_ can be safely neglected for time delays on the picosecond time scale. On the other hand, the term (∂*S*/∂*T*)_ρ_ can be derived by measuring scattering data with enhanced statistics at time delays between 100 and 200 ps and can be used to fit the data. The central panel of Fig. 11 ▸ shows the result of the best fit returned when calculating the solvent term for each individual delay step with Δ*T*(*t*) left as a free parameter. Fig. 12 ▸(*a*) shows the delay dependence of the fit parameters Δ*T*(*t*), with an equilibrium solvent temperature increase of about 3.5° reached with a time constant of ∼6 ps.

The differential scattering signal remaining after subtraction of the solvent term is shown in Fig. 11 ▸(*c*) and is due to the solute and cross (solute–solvent) terms. Structural information of the solute molecule and the rearrangement of the solvent around it can be extracted from these data by computing the time-dependent scattering contributions from excited state structures obtained by model calculations like density functional theory (DFT). However, even in absence of these model calculations, we can extract preliminary information about the solute specific dynamics by integrating the signal within a region of *q* within 0.4 and 0.8 Å^−1^.

The negative feature visible in this range of low *q* values is consistent with Fe—N bond length elongation (Kjaer *et al.*, 2019 ▸). The integrated signal shown in Fig. 12 ▸(*b*) shows also clear evidence of coherent oscillations damped within the first picosecond, with a period of ∼300 fs, which is in excellent agreement with the XANES data presented in Section 3.3.1 (see Fig. 7 ▸).

## 4. Conclusions

This paper demonstrates the capabilities of the Alvra experimental station, which was designed to probe ultrafast structural and electronic dynamics in chemical and biological systems at SwissFEL. Exploiting the use of complementary techniques such as X-ray scattering and spectroscopy, Alvra provides a versatile platform for investigating fundamental processes on femtosecond timescales. During the commissioning phase and first years of user operation, several technical developments have been implemented, including diagnostics for single-shot pulse intensity, position and spectrum as well as improvements to sample delivery, timing tools and detection systems. These improvements have contributed to the generation of a reliable and flexible experimental environment which supports a broad range of scientific applications.

We have reported representative results, focusing on data collected using the cylindrical liquid jet in Alvra Prime. These results highlight the performance and scientific potential of the endstation. These studies showcase the ability of Alvra to capture transient structural intermediates, follow ultrafast chemical reactions, and resolve subtle electronic changes in complex samples, demonstrating that the Alvra experimental station at SwissFEL is one of the world-leading endstations for ultrafast sciences. In addition to its broad experimental flexibility, Alvra distinguishes itself among other XFEL endstations through several unique capabilities. These include access to time-resolved spectroscopies, the tender X-ray regime, enabling studies of light elements and 4*d* transition metal *L*-edges, as well as the possibility to combine multiple spectroscopic and scattering techniques within a single experiment. These capabilities, together with the demonstrated high temporal resolution and optimized sample environments for liquid-phase chemistry, establish Alvra at the same time as a unique and complementary instrument to other XFEL endstations worldwide, particularly for studies of ultrafast chemical and biological dynamics in solution.

## References

[bb1] Auböck, G. & Chergui, M. (2015). *Nat. Chem.* **7**, 629–633. 10.1038/nchem.2305 26201738

[bb2] Bacellar, C., Kinschel, D., Mancini, G. F., Ingle, R. A., Rouxel, J., Cannelli, O., Cirelli, C., Knopp, G., Szlachetko, J., Lima, F. A., Menzi, S., Pamfilidis, G., Kubicek, K., Khakhulin, D., Gawelda, W., Rodriguez-Fernandez, A., Biednov, M., Bressler, C., Arrell, C. A., Johnson, P. J. M., Milne, C. J. & Chergui, M. (2020). *Proc. Natl Acad. Sci. USA* **117**, 21914–21920. 10.1073/pnas.2009490117 PMC7486745 32848065

[bb3] Bacellar, C., Rouxel, J. R., Ingle, R. A., Mancini, G. F., Kinschel, D., Cannelli, O., Zhao, Y., Cirelli, C., Knopp, G., Szlachetko, J., Lima, F. A., Menzi, S., Ozerov, D., Pamfilidis, G., Kubicek, K., Khakhulin, D., Gawelda, W., Rodriguez-Fernandez, A., Biednov, M., Bressler, C., Arrell, C. A., Johnson, P. J. M., Milne, C. J. & Chergui, M. (2023). *J. Phys. Chem. Lett.* **14**, 2425–2432. 10.1021/acs.jpclett.3c00218 36862109

[bb4] Banerjee, A., Jay, R. M., Leitner, T., Wang, R.-P., Harich, J., Stefanuik, R., Coates, M. R., Beale, E. V., Kabanova, V., Kahraman, A., Wach, A., Ozerov, D., Arrell, C., Milne, C., Johnson, P. J. M., Cirelli, C., Bacellar, C., Huse, N., Odelius, M. & Wernet, P. (2024). *Chem. Sci.* **15**, 2398–2409. 10.1039/d3sc04388f PMC10866335 38362433

[bb5] Barends, T. R. M., Gorel, A., Bhattacharyya, S., Schirò, G., Bacellar, C., Cirelli, C., Colletier, J.-P., Foucar, L., Grünbein, M. L., Hartmann, E., Hilpert, M., Holton, J. M., Johnson, P. J. M., Kloos, M., Knopp, G., Marekha, B., Nass, K., Nass Kovacs, G., Ozerov, D., Stricker, M., Weik, M., Doak, R. B., Shoeman, R. L., Milne, C. J., Huix-Rotllant, M., Cammarata, M. & Schlichting, I. (2024). *Nature* **626**, 905–911. 10.1038/s41586-024-07032-9 PMC10881388 38355794

[bb6] Behrens, C., Decker, F.-J., Ding, Y., Dolgashev, V. A., Frisch, J., Huang, Z., Krejcik, P., Loos, H., Lutman, A., Maxwell, T. J., Turner, J., Wang, J., Wang, M.-H., Welch, J. & Wu, J. (2014). *Nat. Commun.* **5**, 3762. 10.1038/ncomms4762 24781868

[bb7] Bettoni, S., Craievich, P., Lutman, A. A. & Pedrozzi, M. (2016). *Phys. Rev. Accel. Beams* **19**, 021304.

[bb8] Bhargava, A., Chen, C. Y., Finkelstein, K. D., Ward, M. J. & Robinson, R. D. (2018). *Phys. Chem. Chem. Phys.* **20**, 28990–29000. 10.1039/c8cp04628j 30238093

[bb9] Bionta, M. R., Hartmann, N., Weaver, M., French, D., Nicholson, D. J., Cryan, J. P., Glownia, J. M., Baker, K., Bostedt, C., Chollet, M., Ding, Y., Fritz, D. M., Fry, A. R., Kane, D. J., Krzywinski, J., Lemke, H. T., Messerschmidt, M., Schorb, S., Zhu, D., White, W. E. & Coffee, R. N. (2014). *Rev. Sci. Instrum.* **85**, 083116. 10.1063/1.4893657 25173255

[bb10] Bionta, M. R., Lemke, H. T., Cryan, J. P., Glownia, J. M., Bostedt, C., Cammarata, M., Castagna, J.-C., Ding, Y., Fritz, D. M., Fry, A. R., Krzywinski, J., Messerschmidt, M., Schorb, S., Swiggers, M. L. & Coffee, R. N. (2011). *Opt. Express* **19**, 21855–21865. 10.1364/OE.19.021855 22109037

[bb11] Borfecchia, E., Garino, C., Salassa, L. & Lamberti, C. (2013). *Philos. Trans. R. Soc. A.* **371**, 20120132. 10.1098/rsta.2012.0132 23776294

[bb12] Bressler, C., Milne, C., Pham, V.-T., Elnahhas, A., van der Veen, R. M., Gawelda, W., Johnson, S., Beaud, P., Grolimund, D., Kaiser, M., Borca, C. N., Ingold, G., Abela, R. & Chergui, M. (2009). *Science* **323**, 489–492. 10.1126/science.1165733 19074309

[bb13] Cammarata, M., Lorenc, M., Kim, T. K., Lee, J. H., Kong, Q. Y., Pontecorvo, E., Lo Russo, M., Schiró, G., Cupane, A., Wulff, M. & Ihee, H. (2006). *J. Chem. Phys.* **124**, 124504. 10.1063/1.2176617 16599694

[bb14] Cannizzo, A., Milne, C., Consani, C., Gawelda, W., Bressler, C., van Mourik, F. & Chergui, M. (2010). *Coord. Chem. Rev.* **254**, 2677–2686.

[bb15] Canton, S. E., Biednov, M., Pápai, M., Lima, F. A., Choi, T.-K., Otte, F., Jiang, Y., Frankenberger, P., Knoll, M., Zalden, P., Gawelda, W., Rahaman, A., Møller, K. B., Milne, C., Gosztola, D. J., Zheng, K., Retegan, M. & Khakhulin, D. (2023). *Adv. Sci.* **10**, 2206880. 10.1002/advs.202206880 PMC10375196 37196414

[bb16] Cellini, A., Shankar, M. K., Nimmrich, A., Hunt, L. A., Monrroy, L., Mutisya, J., Furrer, A., Beale, E. V., Carrillo, M., Malla, T. N., Maj, P., Vrhovac, L., Dworkowski, F., Cirelli, C., Johnson, P. J. M., Ozerov, D., Stojković, E. A., Hammarström, L., Bacellar, C., Standfuss, J., Maj, M., Schmidt, M., Weinert, T., Ihalainen, J. A., Wahlgren, W. Y. & Westenhoff, S. (2024). *Nat. Chem.* **16**, 624–632. 10.1038/s41557-023-01413-9 PMC10997514 38225270

[bb17] Chen, L. X., Rajh, T., Wang, Z. & Thurnauer, M. C. (1997). *J. Phys. Chem. B* **101**, 10688–10697.

[bb18] Chen, L. X., Zhang, X., Wasinger, E. C., Attenkofer, K., Jennings, G., Muresan, A. Z. & Lindsey, J. S. (2007). *J. Am. Chem. Soc.* **129**, 9616–9618. 10.1021/ja072979v 17636917

[bb19] Christou, N.-E., Apostolopoulou, V., Melo, D. V. M., Ruppert, M., Fadini, A., Henkel, A., Sprenger, J., Oberthuer, D., Günther, S., Pateras, A., Rahmani Mashhour, A., Yefanov, O. M., Galchenkova, M., Reinke, P. Y. A., Kremling, V., Scheer, T. E. S., Lange, E. R., Middendorf, P., Schubert, R., De Zitter, E., Lumbao-Conradson, K., Herrmann, J., Rahighi, S., Kunavar, A., Beale, E. V., Beale, J. H., Cirelli, C., Johnson, P. J. M., Dworkowski, F., Ozerov, D., Bertrand, Q., Wranik, M., Bacellar, C., Bajt, S., Wakatsuki, S., Sellberg, J. A., Huse, N., Turk, D., Chapman, H. N. & Lane, T. J. (2023). *Science* **382**, 1015–1020. 10.1126/science.adj4270 38033070

[bb20] Consani, C., Auböck, G., Bräm, O., van Mourik, F. & Chergui, M. (2014). *J. Chem. Phys.* **140**, 025103. 10.1063/1.4861467 24437919

[bb21] David, C., Seniutinas, G., Makita, M., Rösner, B., Rehanek, J., Karvinen, P., Löhl, F., Abela, R., Patthey, L. & Juranić, P. (2021). *J. Synchrotron Rad.* **28**, 1978–1984. 10.1107/S1600577521009619 PMC8570208 34738953

[bb22] de Groot, F. (2001). *Chem. Rev.* **101**, 1779–1808. 10.1021/cr9900681 11709999

[bb23] Dell’Angela, M., Anniyev, T., Beye, M., Coffee, R., Föhlisch, A., Gladh, J., Katayama, T., Kaya, S., Krupin, O., LaRue, J., Møgelhøj, A., Nordlund, D., Nørskov, J. K., Öberg, H., Ogasawara, H., Öström, H., Pettersson, L. G. M., Schlotter, W. F., Sellberg, J. A., Sorgenfrei, F., Turner, J. J., Wolf, M., Wurth, W. & Nilsson, A. (2013). *Science* **339**, 1302–1305. 10.1126/science.1231711 23493709

[bb24] Dijkstal, P., Malyzhenkov, A., Craievich, P., Ferrari, E., Ganter, R., Reiche, S., Schietinger, T., Juranić, P. & Prat, E. (2022). *Phys. Rev. Res.* **4**, 013017. 10.1103/PhysRevLett.123.234801 31868471

[bb25] Ding, Y., Behrens, C., Emma, P., Frisch, J., Huang, Z., Loos, H., Krejcik, P. & Wang, M.-H. (2011). *Phys. Rev. ST Accel. Beams* **14**, 120701.

[bb26] Fadini, A., Reiche, S., Nass, K. & van Thor, J. J. (2020). *Appl. Sci.* **10**, 2599.

[bb27] Follath, R., Flechsig, U., Milne, C., Szlachetko, J., Ingold, G., Patterson, B., Patthey, L. & Abela, R. (2016). *AIP Conf. Proc.* **1741**, 020009.

[bb28] Gaffney, K. J. (2021). *Chem. Sci.* **12**, 8010–8025. 10.1039/d1sc01864g PMC8208315 34194691

[bb29] Garratt, D., Das, S. K., Nelson, K. J., Harich, J., Freibert, A., Bacellar, C., Cirelli, C., Johnson, P. J. M., Castillo, R. G., Zoric, M. R., Wang, R.-P., Lim, H., Cordones, A. A., Huse, N., Odelius, M. & Gaffney, K. (2025). *ChemRxiv*:2025-29gd5.

[bb30] Gawelda, W., Pham, V.-T., Benfatto, M., Zaushitsyn, Y., Kaiser, M., Grolimund, D., Johnson, S. L., Abela, R., Hauser, A., Bressler, C. & Chergui, M. (2007). *Phys. Rev. Lett.* **98**, 057401. 10.1103/PhysRevLett.98.057401 17358897

[bb31] Gruhl, T., Weinert, T., Rodrigues, M. J., Milne, C. J., Ortolani, G., Nass, K., Nango, E., Sen, S., Johnson, P. J. M., Cirelli, C., Furrer, A., Mous, S., Skopintsev, P., James, D., Dworkowski, F., Båth, P., Kekilli, D., Ozerov, D., Tanaka, R., Glover, H., Bacellar, C., Brünle, S., Casadei, C. M., Diethelm, A. D., Gashi, D., Gotthard, G., Guixà-González, R., Joti, Y., Kabanova, V., Knopp, G., Lesca, E., Ma, P., Martiel, I., Mühle, J., Owada, S., Pamula, F., Sarabi, D., Tejero, O., Tsai, C.-J., Varma, N., Wach, A., Boutet, S., Tono, K., Nogly, P., Deupi, X., Iwata, S., Neutze, R., Standfuss, J., Schertler, G. & Panneels, V. (2023). *Nature* **615**, 939–944.

[bb32] Haldrup, K., Christensen, M. & Meedom Nielsen, M. (2010). *Acta Cryst.* A**66**, 261–269. 10.1107/S0108767309054233 20164649

[bb33] Harmand, M., Coffee, R., Bionta, M. R., Chollet, M., French, D., Zhu, D., Fritz, D. M., Lemke, H. T., Medvedev, N., Ziaja, B., Toleikis, S. & Cammarata, M. (2013). *Nat. Photon.* **7**, 215–218.

[bb34] Hauser, A. (2004). In *Spin Crossover in Transition Metal Compounds II*, Vol. 234 of *Topics in Current Chemistry*, pp. 155–198. Berlin, Heidelberg: Springer.

[bb35] Huse, N., Cho, H., Hong, K., Jamula, L., de Groot, F. M. F., Kim, T. K., McCusker, J. K. & Schoenlein, R. W. (2011). *J. Phys. Chem. Lett.* **2**, 880–884. 10.1021/jz200168m 26295622

[bb36] Ihee, H., Wulff, M., Kim, J. & Adachi, S. (2010). *Int. Rev. Phys. Chem.* **29**, 453–520.

[bb37] Jay, R. M., Banerjee, A., Leitner, T., Wang, R.-P., Harich, J., Stefanuik, R., Wikmark, H., Coates, M. R., Beale, E. V., Kabanova, V., Kahraman, A., Wach, A., Ozerov, D., Arrell, C., Johnson, P. J. M., Borca, C. N., Cirelli, C., Bacellar, C., Milne, C., Huse, N., Smolentsev, G., Huthwelker, T., Odelius, M. & Wernet, P. (2023). *Science* **380**, 955–960. 10.1126/science.adf8042 37262165

[bb38] Johnson, P. J. M., Prokhorenko, V. I. & Miller, R. J. D. (2011). *Opt. Lett.* **36**, 2170. 10.1364/OL.36.002170 21633485

[bb39] Juranić, P., Cirelli, C., Mamyrbayev, T., Uemura, Y., Vila–Comamala, J., Lima, F. A., Bacellar, C., Johnson, P. J. M., Prat, E., Reiche, S., Wach, A., Bykova, I., Kahraman, A., Kabanova, V., Milne, C. & David, C. (2024). *Small Methods* **8**, 2301328. 10.1002/smtd.202301328 38441281

[bb40] Juranić, P., Rehanek, J., Arrell, C. A., Pradervand, C., Ischebeck, R., Erny, C., Heimgartner, P., Gorgisyan, I., Thominet, V., Tiedtke, K., Sorokin, A., Follath, R., Makita, M., Seniutinas, G., David, C., Milne, C. J., Lemke, H., Radovic, M., Hauri, C. P. & Patthey, L. (2018). *J. Synchrotron Rad.* **25**, 1238–1248. 10.1107/S1600577518005775 PMC6038612 29979187

[bb41] Kabanova, V., Sander, M., Levantino, M., Kong, Q., Canton, S., Retegan, M., Cammarata, M., Lenzen, P., Lawson, L. M. D. & Wulff, M. (2024). *Struct. Dyn.* **11**, 054901. 10.1063/4.0000254 PMC11501788 39449690

[bb42] Kim, Y., Nam, D., Ma, R., Kim, S., Kim, M.-J., Kim, J., Eom, I., Lee, J. H. & Kim, T. K. (2022). *J. Synchrotron Rad.* **29**, 194–201. 10.1107/S1600577521011449 PMC8733995 34985436

[bb43] Kirkpatrick, P. & Baez, A. V. (1948). *J. Opt. Soc. Am.* **38**, 766–774. 10.1364/josa.38.000766 18883922

[bb44] Kjaer, K. S., Kunnus, K., Harlang, T. C. B., Van Driel, T. B., Ledbetter, K., Hartsock, R. W., Reinhard, M. E., Koroidov, S., Li, L., Laursen, M. G., Biasin, E., Hansen, F. B., Vester, P., Christensen, M., Haldrup, K., Nielsen, M. M., Chabera, P., Liu, Y., Tatsuno, H., Timm, C., Uhlig, J., Sundstöm, V., Németh, Z., Szemes, D. S., Bajnóczi, É., Vankó, G., Alonso-Mori, R., Glownia, J. M., Nelson, S., Sikorski, M., Sokaras, D., Lemke, H. T., Canton, S. E., Wärnmark, K., Persson, P., Cordones, A. A. & Gaffney, K. J. (2018). *Phys. Chem. Chem. Phys.* **20**, 4238–4249. 10.1039/c7cp07838b 29364300

[bb45] Kjaer, K. S., Van Driel, T. B., Harlang, T. C. B., Kunnus, K., Biasin, E., Ledbetter, K., Hartsock, R. W., Reinhard, M. E., Koroidov, S., Li, L., Laursen, M. G., Hansen, F. B., Vester, P., Christensen, M., Haldrup, K., Nielsen, M. M., Dohn, A. O., Pápai, M. I., Møller, K. B., Chabera, P., Liu, Y., Tatsuno, H., Timm, C., Jarenmark, M., Uhlig, J., Sundstöm, V., Wärnmark, K., Persson, P., Németh, Z., Szemes, D. S., Bajnóczi, É., Vankó, G., Alonso-Mori, R., Glownia, J. M., Nelson, S., Sikorski, M., Sokaras, D., Canton, S. E., Lemke, H. T. & Gaffney, K. J. (2019). *Chem. Sci.* **10**, 5749–5760. 10.1039/c8sc04023k PMC6568243 31293761

[bb46] Kjær, K. S., van Driel, T. B., Kehres, J., Haldrup, K., Khakhulin, D., Bechgaard, K., Cammarata, M., Wulff, M., Sørensen, T. J. & Nielsen, M. M. (2013). *Phys. Chem. Chem. Phys.* **15**, 15003–15016. 10.1039/c3cp50751c 23918050

[bb47] Kong, Q., Lee, J. H., Kim, K. H., Kim, J., Wulff, M., Ihee, H. & Koch, M. H. J. (2010). *J. Am. Chem. Soc.* **132**, 2600–2607. 10.1021/ja9097548 20141137

[bb48] Kunnus, K., Zhang, W., Delcey, M. G., Pinjari, R. V., Miedema, P. S., Schreck, S., Quevedo, W., Schröder, H., Föhlisch, A., Gaffney, K. J., Lundberg, M., Odelius, M. & Wernet, P. (2016). *J. Phys. Chem. B* **120**, 7182–7194. 10.1021/acs.jpcb.6b04751 27380541

[bb49] Lemke, H. T., Bressler, C., Chen, L. X., Fritz, D. M., Gaffney, K. J., Galler, A., Gawelda, W., Haldrup, K., Hartsock, R. W., Ihee, H., Kim, J., Kim, K. H., Lee, J. H., Nielsen, M. M., Stickrath, A. B., Zhang, W., Zhu, D. & Cammarata, M. (2013). *J. Phys. Chem. A* **117**, 735–740. 10.1021/jp312559h 23281652

[bb50] Lemke, H. T., Kjaer, K. S., Hartsock, R., van Driel, T. B., Chollet, M., Glownia, J. M., Song, S., Zhu, D., Pace, E., Matar, S. F., Nielsen, M. M., Benfatto, M., Gaffney, K. J., Collet, E. & Cammarata, M. (2017). *Nat. Commun.* **8**, 15342. 10.1038/ncomms15342 PMC5458100 28537270

[bb51] Li, N., Bediako, D. K., Hadt, R. G., Hayes, D., Kempa, T. J., von Cube, F., Bell, D. C., Chen, L. X. & Nocera, D. G. (2017). *Proc. Natl Acad. Sci. USA* **114**, 1486–1491. 10.1073/pnas.1620787114 PMC5321006 28137835

[bb52] Lim, H., Yang, X., Larsen, C. B., Ledbetter, K., Zoric, M. R., Raj, S. L., Kumar, G., Powers-Riggs, N., Hoffmann, M. C., Chollet, M., Gee, L. B., van Driel, T. B., Alonso-Mori, R., Kabanova, V., Kahraman, A., Johnson, P. J. M., Cirelli, C., Bacellar, C., Gaffney, K. J., Li, X. & Cordones, A. A. (2025). *J. Am. Chem. Soc.* **147**, 7496–7506. 10.1021/jacs.4c16212 39993950

[bb54] Löhl, F., Arsov, V., Felber, M., Hacker, K., Jalmuzna, W., Lorbeer, B., Ludwig, F., Matthiesen, K.-H., Schlarb, H., Schmidt, B., Schmüser, P., Schulz, S., Szewinski, J., Winter, A. & Zemella, J. (2010). *Phys. Rev. Lett.* **104**, 144801. 10.1103/PhysRevLett.104.144801 20481941

[bb53] Lutman, A. A., Maxwell, T. J., MacArthur, J. P., Guetg, M. W., Berrah, N., Coffee, R. N., Ding, Y., Huang, Z., Marinelli, A., Moeller, S. & Zemella, J. C. U. (2016). *Nat. Photon.* **10**, 745–750.

[bb55] Mara, M. W., Hadt, R. G., Reinhard, M. E., Kroll, T., Lim, H., Hartsock, R. W., Alonso-Mori, R., Chollet, M., Glownia, J. M., Nelson, S., Sokaras, D., Kunnus, K., Hodgson, K. O., Hedman, B., Bergmann, U., Gaffney, K. J. & Solomon, E. I. (2017). *Science* **356**, 1276–1280. 10.1126/science.aam6203 PMC5706643 28642436

[bb56] Meisel, A., Leonhardt, G. & Szargan, R. (1989). *X-ray Spectra and Chemical Binding*, Vol. 37 of *Springer Series in Chemical Physics.* Berlin, Heidelberg: Springer.

[bb57] Milne, C., Radi, P., Pedrini, B., Lemke, H., Knopp, G., Juranic, P., Ingold, G., Hauri, C. P., Flechsig, U., Follath, R., Erny, C., Deng, Y., Beaud, P. & Patthey, L. (2017*b*). *Chimia* **71**, 299–307. 10.2533/chimia.2017.299 28576157

[bb58] Milne, C., Schietinger, T., Aiba, M., Alarcon, A., Alex, J., Anghel, A., Arsov, V., Beard, C., Beaud, P., Bettoni, S., Bopp, M., Brands, H., Brönnimann, M., Brunnenkant, I., Calvi, M., Citterio, A., Craievich, P., Csatari Divall, M., Dällenbach, M., D’Amico, M., Dax, A., Deng, Y., Dietrich, A., Dinapoli, R., Divall, E., Dordevic, S., Ebner, S., Erny, C., Fitze, H., Flechsig, U., Follath, R., Frei, F., Gärtner, F., Ganter, R., Garvey, T., Geng, Z., Gorgisyan, I., Gough, C., Hauff, A., Hauri, C., Hiller, N., Humar, T., Hunziker, S., Ingold, G., Ischebeck, R., Janousch, M., Juranić, P., Jurcevic, M., Kaiser, M., Kalantari, B., Kalt, R., Keil, B., Kittel, C., Knopp, G., Koprek, W., Lemke, H., Lippuner, T., Llorente Sancho, D., Löhl, F., Lopez-Cuenca, C., Märki, F., Marcellini, F., Marinkovic, G., Martiel, I., Menzel, R., Mozzanica, A., Nass, K., Orlandi, G., Ozkan Loch, C., Panepucci, E., Paraliev, M., Patterson, B., Pedrini, B., Pedrozzi, M., Pollet, P., Pradervand, C., Prat, E., Radi, P., Raguin, J., Redford, S., Rehanek, J., Réhault, J., Reiche, S., Ringele, M., Rittmann, J., Rivkin, L., Romann, A., Ruat, M., Ruder, C., Sala, L., Schebacher, L., Schilcher, T., Schlott, V., Schmidt, T., Schmitt, B., Shi, X., Stadler, M., Stingelin, L., Sturzenegger, W., Szlachetko, J., Thattil, D., Treyer, D., Trisorio, A., Tron, W., Vetter, S., Vicario, C., Voulot, D., Wang, M., Zamofing, T., Zellweger, C., Zennaro, R., Zimoch, E., Abela, R., Patthey, L. & Braun, H. (2017*a*). *Appl. Sci.* **7**, 720.

[bb59] Mous, S., Gotthard, G., Ehrenberg, D., Sen, S., Weinert, T., Johnson, P. J. M., James, D., Nass, K., Furrer, A., Kekilli, D., Ma, P., Brünle, S., Casadei, C. M., Martiel, I., Dworkowski, F., Gashi, D., Skopintsev, P., Wranik, M., Knopp, G., Panepucci, E., Panneels, V., Cirelli, C., Ozerov, D., Schertler, G. F. X., Wang, M., Milne, C., Standfuss, J., Schapiro, I., Heberle, J. & Nogly, P. (2022). *Science* **375**, 845–851. 10.1126/science.abj6663 35113649

[bb60] Mozzanica, A., Andrä, M., Barten, R., Bergamaschi, A., Chiriotti, S., Brückner, M., Dinapoli, R., Fröjdh, E., Greiffenberg, D., Leonarski, F., Lopez-Cuenca, C., Mezza, D., Redford, S., Ruder, C., Schmitt, B., Shi, X., Thattil, D., Tinti, G., Vetter, S. & Zhang, J. (2018). *Synchrotron Radiat. News* **31**(6), 16–20.

[bb61] Nass, K., Bacellar, C., Cirelli, C., Dworkowski, F., Gevorkov, Y., James, D., Johnson, P. J. M., Kekilli, D., Knopp, G., Martiel, I., Ozerov, D., Tolstikova, A., Vera, L., Weinert, T., Yefanov, O., Standfuss, J., Reiche, S. & Milne, C. J. (2021). *IUCrJ* **8**, 905–920. 10.1107/S2052252521008046 PMC8562661 34804544

[bb82] Öström, H., Öberg, H., Xin, H., LaRue, J., Beye, M., Dell’Angela, M., Gladh, J., Ng, M. L., Sellberg, J. A., Kaya, S., Mercurio, G., Nordlund, D., Hantschmann, M., Hieke, F., Kühn, D., Schlotter, W. F., Dakovski, G. L., Turner, J. J., Minitti, M. P., Mitra, A., Moeller, S. P., Föhlisch, A., Wolf, M., Wurth, W., Persson, M., Nørskov, J. K., Abild-Pedersen, F., Ogasawara, H., Pettersson, L. G. M. & Nilsson, A. (2015). *Science* **347**, 978–982. 10.1126/science.1261747 25722407

[bb62] Prat, E., Abela, R., Aiba, M., Alarcon, A., Alex, J., Arbelo, Y., Arrell, C., Arsov, V., Bacellar, C., Beard, C., Beaud, P., Bettoni, S., Biffiger, R., Bopp, M., Braun, H., Calvi, M., Cassar, A., Celcer, T., Chergui, M., Chevtsov, P., Cirelli, C., Citterio, A., Craievich, P., Divall, M. C., Dax, A., Dehler, M., Deng, Y., Dietrich, A., Dijkstal, P., Dinapoli, R., Dordevic, S., Ebner, S., Engeler, D., Erny, C., Esposito, V., Ferrari, E., Flechsig, U., Follath, R., Frei, F., Ganter, R., Garvey, T., Geng, Z., Gobbo, A., Gough, C., Hauff, A., Hauri, C. P., Hiller, N., Hunziker, S., Huppert, M., Ingold, G., Ischebeck, R., Janousch, M., Johnson, P. J. M., Johnson, S. L., Juranić, P., Jurcevic, M., Kaiser, M., Kalt, R., Keil, B., Kiselev, D., Kittel, C., Knopp, G., Koprek, W., Laznovsky, M., Lemke, H. T., Sancho, D. L., Löhl, F., Malyzhenkov, A., Mancini, G. F., Mankowsky, R., Marcellini, F., Marinkovic, G., Martiel, I., Märki, F., Milne, C. J., Mozzanica, A., Nass, K., Orlandi, G. L., Loch, C. O., Paraliev, M., Patterson, B., Patthey, L., Pedrini, B., Pedrozzi, M., Pradervand, C., Radi, P., Raguin, J., Redford, S., Rehanek, J., Reiche, S., Rivkin, L., Romann, A., Sala, L., Sander, M., Schietinger, T., Schilcher, T., Schlott, V., Schmidt, T., Seidel, M., Stadler, M., Stingelin, L., Svetina, C., Treyer, D. M., Trisorio, A., Vicario, C., Voulot, D., Wrulich, A., Zerdane, S. & Zimoch, E. (2020). *Nat. Photon.* **14**, 748–754.

[bb63] Rehanek, J., Makita, M., Wiegand, P., Heimgartner, P., Pradervand, C., Seniutinas, G., Flechsig, U., Thominet, V., Schneider, C., Fernandez, A. R., David, C., Patthey, L. & Juranić, P. (2017). *J. Instrum.* **12**, P05024.

[bb64] Reiche, S., Bacellar, C., Bougiatioti, P., Cirelli, C., Dijkstal, P., Ferrari, E., Juranić, P., Knopp, G., Malyzhenkov, A., Milne, C., Nass, K., Prat, E., Vila-Comamala, J. & David, C. (2023). *Phys. Rev. Res.* **5**, L022009.

[bb65] Saes, M., Bressler, C., Abela, R., Grolimund, D., Johnson, S. L., Heimann, P. A. & Chergui, M. (2003). *Phys. Rev. Lett.* **90**, 047403. 10.1103/PhysRevLett.90.047403 12570459

[bb66] Scholes, G. D., Fleming, G. R., Chen, L. X., Aspuru-Guzik, A., Buchleitner, A., Coker, D. F., Engel, G. S., van Grondelle, R., Ishizaki, A., Jonas, D. M., Lundeen, J. S., McCusker, J. K., Mukamel, S., Ogilvie, J. P., Olaya-Castro, A., Ratner, M. A., Spano, F. C., Whaley, K. B. & Zhu, X. (2017). *Nature* **543**, 647–656. 10.1038/nature21425 28358065

[bb67] Skopintsev, P., Ehrenberg, D., Weinert, T., James, D., Kar, R. K., Johnson, P. J. M., Ozerov, D., Furrer, A., Martiel, I., Dworkowski, F., Nass, K., Knopp, G., Cirelli, C., Arrell, C., Gashi, D., Mous, S., Wranik, M., Gruhl, T., Kekilli, D., Brünle, S., Deupi, X., Schertler, G. F. X., Benoit, R. M., Panneels, V., Nogly, P., Schapiro, I., Milne, C., Heberle, J. & Standfuss, J. (2020). *Nature* **583**, 314–318. 10.1038/s41586-020-2307-8 32499654

[bb68] Sweetser, J. N., Fittinghoff, D. N. & Trebino, R. (1997). *Opt. Lett.* **22**, 519. 10.1364/ol.22.000519 18183253

[bb69] Szlachetko, J., Nachtegaal, M., de Boni, E., Willimann, M., Safonova, O., Sa, J., Smolentsev, G., Szlachetko, M., van Bokhoven, J. A., Dousse, J.-C., Hoszowska, J., Kayser, Y., Jagodzinski, P., Bergamaschi, A., Schmitt, B., David, C. & Lücke, A. (2012). *Rev. Sci. Instrum.* **83**, 103105. 10.1063/1.4756691 23126749

[bb70] Tono, K., Kudo, T., Yabashi, M., Tachibana, T., Feng, Y., Fritz, D., Hastings, J. & Ishikawa, T. (2011). *Rev. Sci. Instrum.* **82**, 023108. 10.1063/1.3549133 21361574

[bb71] Urch, D. (1971). *Q. Rev. Chem. Soc.* **25**, 343–364.

[bb72] Vankó, G., Bordage, A., Glatzel, P., Gallo, E., Rovezzi, M., Gawelda, W., Galler, A., Bressler, C., Doumy, G., March, A. M., Kanter, E. P., Young, L., Southworth, S. H., Canton, S. E., Uhlig, J., Smolentsev, G., Sundström, V., Haldrup, K., van Driel, T. B., Nielsen, M. M., Kjaer, K. S. & Lemke, H. T. (2013). *J. Electron Spectrosc. Relat. Phenom.* **188**, 166–171.

[bb73] Vankó, G., Neisius, T., Molnár, G., Renz, F., KÁRPáti, S., Shukla, A. & de Groot, F. M. F. (2006). *J. Phys. Chem. B* **110**, 11647–11653. 10.1021/jp0615961 16800459

[bb74] Weierstall, U., James, D., Wang, C., White, T. A., Wang, D., Liu, W., Spence, J. C. H., Bruce Doak, R., Nelson, G., Fromme, P., Fromme, R., Grotjohann, I., Kupitz, C., Zatsepin, N. A., Liu, H., Basu, S., Wacker, D., Won Han, G., Katritch, V., Boutet, S., Messerschmidt, M., Williams, G. J., Koglin, J. E., Marvin Seibert, M., Klinker, M., Gati, C., Shoeman, R. L., Barty, A., Chapman, H. N., Kirian, R. A., Beyerlein, K. R., Stevens, R. C., Li, D., Shah, S. T. A., Howe, N., Caffrey, M. & Cherezov, V. (2014). *Nat. Commun.* **5**, 3309. 10.1038/ncomms4309 PMC4061911 24525480

[bb75] Wernet, P., Kunnus, K., Josefsson, I., Rajkovic, I., Quevedo, W., Beye, M., Schreck, S., Grübel, S., Scholz, M., Nordlund, D., Zhang, W., Hartsock, R. W., Schlotter, W. F., Turner, J. J., Kennedy, B., Hennies, F., de Groot, F. M. F., Gaffney, K. J., Techert, S., Odelius, M. & Föhlisch, A. (2015). *Nature* **520**, 78–81. 10.1038/nature14296 25832405

[bb76] Wranik, M., Weinert, T., Slavov, C., Masini, T., Furrer, A., Gaillard, N., Gioia, D., Ferrarotti, M., James, D., Glover, H., Carrillo, M., Kekilli, D., Stipp, R., Skopintsev, P., Brünle, S., Mühlethaler, T., Beale, J., Gashi, D., Nass, K., Ozerov, D., Johnson, P. J. M., Cirelli, C., Bacellar, C., Braun, M., Wang, M., Dworkowski, F., Milne, C., Cavalli, A., Wachtveitl, J., Steinmetz, M. O. & Standfuss, J. (2023). *Nat. Commun.* **14**, 903. 10.1038/s41467-023-36481-5 PMC9936131 36807348

[bb77] Xie, X., Deng, Y. & Johnson, S. L. (2021). *High Power Laser Sci. Eng.* **9**, e66.

[bb78] Xie, X., Hung, Y., Deng, Y., Cavalieri, A. L., Baltuška, A. & Johnson, S. L. (2024). *High Power Laser Sci. Eng.* **12**, e16.

[bb79] Yano, J. & Yachandra, V. K. (2009). *Photosynth. Res.* **102**, 241–254. 10.1007/s11120-009-9473-8 PMC2777224 19653117

[bb80] Yong, H., Xu, X., Ruddock, J. M., Stankus, B., Carrascosa, A. M., Zotev, N., Bellshaw, D., Du, W., Goff, N., Chang, Y., Boutet, S., Carbajo, S., Koglin, J. E., Liang, M., Robinson, J. S., Kirrander, A., Minitti, M. P. & Weber, P. M. (2021). *Proc. Natl Acad. Sci. USA* **118**, e2021714118. 10.1073/pnas.2021714118 PMC8126834 33947814

[bb81] Zhang, W., Alonso-Mori, R., Bergmann, U., Bressler, C., Chollet, M., Galler, A., Gawelda, W., Hadt, R. G., Hartsock, R. W., Kroll, T., Kjaer, K. S., Kubiček, K., Lemke, H. T., Liang, H. W., Meyer, D. A., Nielsen, M. M., Purser, C., Robinson, J. S., Solomon, E. I., Sun, Z., Sokaras, D., van Driel, T. B., Vankó, G., Weng, T.-C., Zhu, D. & Gaffney, K. J. (2014). *Nature* **509**, 345–348. 10.1038/nature13252 PMC5668134 24805234

